# Improved accuracy of cerebral blood flow quantification in the presence of systemic physiology cross-talk using multi-layer Monte Carlo modeling

**DOI:** 10.1117/1.NPh.8.1.015001

**Published:** 2021-01-01

**Authors:** Melissa M. Wu, Suk-Tak Chan, Dibbyan Mazumder, Davide Tamborini, Kimberly A. Stephens, Bin Deng, Parya Farzam, Joyce Yawei Chu, Maria Angela Franceschini, Jason Zhensheng Qu, Stefan A. Carp

**Affiliations:** a Massachusetts General Hospital, Harvard Medical School, Optics at Athinoula A. Martinos Center for Biomedical Imaging, Department of Radiology, Charlestown, Massachusetts, United States; b Massachusetts General Hospital, Harvard Medical School, Department of Anesthesia, Critical Care and Pain Medicine, Boston, Massachusetts, United States

**Keywords:** diffuse correlation spectroscopy, Monte Carlo, hypercapnia, cerebral blood flow, multi-layer

## Abstract

**Significance:** Contamination of diffuse correlation spectroscopy (DCS) measurements of cerebral blood flow (CBF) due to systemic physiology remains a significant challenge in the clinical translation of DCS for neuromonitoring. Tunable, multi-layer Monte Carlo-based (MC) light transport models have the potential to remove extracerebral flow cross-talk in cerebral blood flow index (${\mathrm{CBF}}_{i}$) estimates.

**Aim:** We explore the effectiveness of MC DCS models in recovering accurate ${\mathrm{CBF}}_{i}$ changes in the presence of strong systemic physiology variations during a hypercapnia maneuver.

**Approach:** Multi-layer slab and head-like realistic (curved) geometries were used to run MC simulations of photon propagation through the head. The simulation data were post-processed into models with variable extracerebral thicknesses and used to fit DCS multi-distance intensity autocorrelation measurements to estimate ${\mathrm{CBF}}_{i}$ timecourses. The results of the MC ${\mathrm{CBF}}_{i}$ values from a set of human subject hypercapnia sessions were compared with ${\mathrm{CBF}}_{i}$ values estimated using a semi-infinite analytical model, as commonly used in the field.

**Results:** Group averages indicate a gradual systemic increase in blood flow following a different temporal profile versus the expected rapid CBF response. Optimized MC models, guided by several intrinsic criteria and a pressure modulation maneuver, were able to more effectively separate ${\mathrm{CBF}}_{i}$ changes from scalp blood flow influence than the analytical fitting, which assumed a homogeneous medium. Three-layer models performed better than two-layer ones; slab and curved models achieved largely similar results, though curved geometries were closer to physiological layer thicknesses.

**Conclusion:** Three-layer, adjustable MC models can be useful in separating distinct changes in scalp and brain blood flow. Pressure modulation, along with reasonable estimates of physiological parameters, can help direct the choice of appropriate layer thicknesses in MC models.

## 1 Introduction

The brain receives 12% to 15% of cardiac output even though it weighs only 2% of the body weight.^1^^,^^2^ Cerebral blood flow (CBF) is responsible for brain oxygen delivery, and thus accurate, continuous CBF quantification can provide crucial information for monitoring brain health and function.^3^^,^^4^ This is particularly important under conditions when cerebral autoregulation may be impaired, potentially leading to insufficient blood flow to the brain.^4^^,^^5^ CBF can be a useful biomarker for both diagnosing and managing patients suffering from stroke, traumatic brain injury, or other neurological impairments.^6^ Therefore, a monitoring technique able to continuously measure CBF non-invasively is highly desirable.

Diffuse correlation spectroscopy (DCS) is becoming increasingly widespread as a non-invasive optical technology to measure tissue perfusion, particularly in the brain.^3^^,^^7^ A long coherence-length laser emitting light in the near-infrared range is used to illuminate the probed tissue region, and photon counting detectors are used to detect speckle fluctuations in the light; the temporal autocorrelation of these fluctuations in the reflected light can then be used to characterize the motion of light scatterers in the medium, in this case red blood cells.^5^^,^^8^ DCS takes advantage of this physical phenomenon to monitor time-varying blood flow non-invasively and is currently being used in various research applications.^5^^,^^8^^–^^15^ A particularly successful application area is the CBF monitoring of neonates, for whom the relatively thin skull results in high brain sensitivity.^16^^,^^17^

Extending the application of DCS to bedside brain monitoring of adults, however, requires accounting for the influence of systemic variations of scalp blood flow (SBF) that have the potential to strongly contaminate changes in the calculated cerebral blood flow index (${\mathrm{CBF}}_{i}$).^3^^,^^18^^,^^19^ Due to the thicker scalp and skull of an adult head, less photons reach the brain, which results in decreased cerebral sensitivity. This further increases the contribution of extracerebral hemodynamic changes in the computed ${\mathrm{CBF}}_{i}$. To extract ${\mathrm{CBF}}_{i}$ measures, most publications in the field primarily use the analytical solution of the diffusion correlation equation—in this paper, referred to as the “analytical model” or “analytical fit”—and assume a single-layer, homogeneous slab medium as the human head.^7^^,^^15^^,^^16^^,^^19^^,^^20^ While scalp influence on brain measurements in other optical neuromonitoring modalities such as near-infrared spectroscopy has been extensively explored and addressed,^21^^–^^26^ development of robust methodologies of separating skin from brain blood flow in DCS is still an ongoing investigation.^3^

Previous works have shown success in using two- and three-layered models to extract ${\mathrm{CBF}}_{i}$ from SBF contamination.^18^^,^^27^^,^^28^ Many of these studies derive a layered adaption of the analytical model, and subsequently use it to fit for and compare quantities averaged into single values representing ${\mathrm{CBF}}_{i}$ before and during an intervention or stimulus, such as hypocapnia or a finger-tapping task. To be used as a potential clinical technology, however, DCS must be able to continuously quantify accurate ${\mathrm{CBF}}_{i}$ changes such that appropriate and timely treatment can be administered. In particular, Baker et al.^29^ have demonstrated the efficacy of a pressure modulation algorithm, where light pressure is applied to occlude SBF for a brief period of time. They combine this with a two-layer analytical model to separate scalp and CBF during a continuous timecourse.

Monte Carlo (MC) fitting methods involve fitting for blood flow with a model based on an MC forward simulation that is equivalent to modeling photon transport through the more accurate radiative transfer equation as opposed to the diffusion correlation equation, which is based on diffusion approximations.^30^ The MC simulations can model light transport in arbitrarily complex 3-D volumes and output photon propagation metrics for every detected photon. For DCS, photon pathlengths and accumulated momentum transfer can be used to compute the temporal field autocorrelation function in fitting for blood flow.^31^ MC fitting methods can be advantageous compared to analytical methods for several reasons. First, as Li et al.^32^ have demonstrated, MC models can accommodate different geometries, allowing surface curvature effects to be investigated on DCS measurements. Second, MC simulations are more accurate at the short detector separations needed to get scalp-specific measurements, separations that test the limits of the diffuse light propagation approximations. Last, MC simulations results are more robust compared to multi-layer analytical models that depend on potentially unstable numerical integrations. Many published DCS works utilize the blood flow index (${\mathrm{BF}}_{i}$) calculated from MC forward simulations as an *in-silico* verification or investigation prior to performing phantom or *in-vivo* studies.^32^^–^^35^ In contrast, taking advantage of advances in computational power and in particular GPU acceleration, we use MC simulations directly as the forward component of our inverse model for estimating ${\mathrm{BF}}_{i}$ in both superficial (scalp) and deep (brain) tissue layers.

This paper aims to evaluate the effectiveness of MC fitting methods in removing extracerebral contaminants from DCS ${\mathrm{CBF}}_{i}$ estimates. For the forward simulations, we employ tunable multi-layer, heterogeneous slab and realistic (head-like) models, the latter of which is derived from an atlas structural MRI scan of a generic human head.^33^ We then allow the model parameters to be adjusted as needed on a case-by-case basis. Both the analytical and MC-based models are used to fit DCS data from an *in-vivo* study on healthy volunteers, during which a pressure modulation maneuver was first performed on each subject before a CBF increase was induced with carbon dioxide administration. A subset of the data was also acquired concurrently with transcranial Doppler ultrasound (TCD) as a validation metric to measure the middle cerebral artery velocity (MCAV). We apply MC-based models, tuned based on several criteria, to extract ${\mathrm{CBF}}_{i}$ in subjects where the hypercapnia-induced significant elevations in SBF. Improvements between the MC ${\mathrm{CBF}}_{i}$ versus the analytical semi-infinite medium solution to the diffusion correlation equation are then evaluated by comparing each timecourse to what we observe with TCD MCAV. To conclude, we discuss the relative performance and advantages among the various MC models used, and the limitations of MC-based fitting.

## 2 Methods

### 2.1 Hypercapnia Study in Healthy Volunteers

We conducted a human subject study with healthy volunteers to measure cerebral responses to induce hypercapnia. As part of a larger hypercapnia challenge study that had several goals beyond the scope of this paper, we enrolled a total of 27 subjects, each of which had either one or two visits, i.e. sessions. The first visit consisted of either optical-only or joint optical-TCD measurements, and the second consisted of joint optical-MRI scanning measurements; in total, 43 measurement sessions were held. Seventeen of the subjects had an MRI scanning session and 9 of the subjects had joint optical-TCD measurements, where TCD data were acquired concurrently with the optical recordings. A pressure modulation maneuver was also performed on each subject before the hypercapnia session. The study was approved by the Mass General Brigham Institutional Review Board, and all subjects provided informed consent prior to the measurements. A table of the subject information is shown in Table 1.

**Table 1:** Subject information. Values for age, baseline ${\mathrm{PetCO}}_{2}$, and increase in ${\mathrm{PetCO}}_{2}$ are given as mean±standard deviation.

Subject information	
Total number of subjects	27
Total number of female subjects	19
Total number of male subjects	8
Average age (years)	26±5
Average baseline $P_{\mathrm{ET}}{\mathrm{CO}}_{2}$ (mmHg)	36±4
Average increase in $P_{\mathrm{ET}}{\mathrm{CO}}_{2}$ (mmHg)	11±1
Subject information	
Total number of sessions	43
Total number of optical-only sessions	17
Total number of optical-MRI sessions	17
Total number of optical-TCD sessions	9

#### 2.1.1 Instrumentation

An in-house gas delivery and mixing system comprising of a medical gas mixer in series with a manifold of flow meters was used to mix and deliver a gas mixture for an exogenous ${\mathrm{CO}}_{2}$ challenge. Given that there is significant inter-individual variance in resting end-tidal carbon dioxide ($P_{\mathrm{ET}}{\mathrm{CO}}_{2}$),^36^ resting $P_{\mathrm{ET}}{\mathrm{CO}}_{2}$ was assessed in subjects via calibrated capnograph before the exogenous ${\mathrm{CO}}_{2}$ challenge. The subject wore a nose-clip and breathed through a mouth-piece on an MRI-compatible circuit designed to maintain the $P_{\mathrm{ET}}{\mathrm{CO}}_{2}$ within ±1 to 2 mmHg of target $P_{\mathrm{ET}}{\mathrm{CO}}_{2}$.^37^^,^^38^ The partial pressures of ${\mathrm{CO}}_{2}$ and $O_{2}$ were sampled through the air filter connected with the mouthpiece, and the sampled gases were measured by ${\mathrm{CO}}_{2}$ and $O_{2}$ gas analyzers (Capstar-100, Oxystar-100, CWE, Inc., Pennsylvania). Both the gas analyzers were again calibrated to the barometric pressure on the day of the experiment and corrected for vapor pressure. The respiratory flow was measured with a respiratory flow head (MTL300L, ADInstruments, Inc., Colorado) on the breathing circuit via a calibrated differential pressure sensor. Physiological changes including ${\mathrm{PCO}}_{2}$, ${\mathrm{PO}}_{2}$, respiration, and ECG were recorded using AdInstruments Powerlab (AdInstruments, Inc., Colorado).

One or two of several available 4-channel continuous-wave DCS instruments built by our group were used to acquire data for each measurement (as constrained by instrument sharing logistics and some technical failures). Each instrument contained a long-coherence length laser, 4 single photon counting avalanche photo diodes (Excelitas SPCM-AQRH or SPCM-AQ4C), and a custom-designed time-tagging board that was used to stream the photon detection timestamps to a laptop computer for recording and display. The wavelengths of the lasers differed across the instruments and were either 767 nm (5 sessions), 785 nm (1 session), or 850 nm (37 sessions). However, as shown in Table 2, the effective attenuation coefficient varies less than 10% between these wavelengths, and so the variation in the average photon penetration and corresponding brain sensitivity was likely small compared to the variation between subjects.

**Table 2:** Optical properties used for each wavelength.

	$\mu_{a}$ (${\mathrm{mm}}^{-1}$)	$\mu_{s}^{\prime }$ (${\mathrm{mm}}^{-1}$)	$\mu_{\mathrm{eff}}$ (${\mathrm{mm}}^{-1}$)
Forehead			
767 nm	0.018	0.933	0.224
785 nm	0.016	0.901	0.208
850 nm	0.020	0.800	0.219
Calf			
785 nm	0.016	0.570	0.165
850 nm	0.018	0.530	0.169

We used DCS custom optical probes with source–detector (s–d) distances of 5, 25, and 30 mm, which were placed on the subject’s forehead. If a single 4-channel instrument was used, we recorded using one fiber at 5 mm, and three fibers bundled together at 30 mm. If a second instrument was available, we also recorded with three bundled fibers at 25-mm separation. In 10 subjects undergoing a total of 16 measurement sessions, a separate probe with a single fiber at a 20-mm s–d distance was attached on the leg of the subject to monitor peripheral blood flow changes. The goal of using of three single-mode fibers bundled together at the 25- and 30-mm distances was to increase signal-to-noise ratio. Prisms were used to couple light from the fibers to the tissue, and a diffuser was placed in front of the 400-μm multi-mode source fiber to ensure ANSI skin laser exposure limits were observed.

In some subjects, a dual probe setting with 2-MHz transducers in conjunction with a TCD system (Delicate EMS-9U, Shenzhen, China) was used for simultaneous recording of cerebral blood flow velocity (CBFV) in the middle cerebral artery (MCA) on both left and right sides. Two transducers were attached to the left and right temporal bone windows by velcro. The depth of the Doppler samples was confined to the M1 segment, which is at the main stem of the MCA. In these measurements, we aimed to capture MCAV timecourses for the left and right middle cerebral arteries (LMCA and RMCA), although in certain cases signal from only one side was able to be acquired. The CBFV was sampled at the rate of 100 Hz, and a trigger was used to align the starts of the TCD, DCS, and respiratory measurements.

#### 2.1.2 Pressure modulation and hypercapnia protocol

A session started with each subject positioned either in a reclined sitting posture or lying down in a supine position. They were asked to bite into the mouthpiece of the breathing circuit for gas administration and sampling, and the subject’s nose was sealed with a clip or tape to ensure accurate gas delivery and sampling. The main DCS probe was placed on the right side of the subject forehead. In a subset of the cases, a probe containing a 20-mm s–d distance was taped to the subject’s calf to measure any potential systemic response in blood flow during hypercapnia. For cases where validation data were also acquired, TCD probes were placed on each temple of the forehead.

Before gas administration, two pressure modulation measurements were taken first. Each consisted of a 20-s baseline period followed by a 30-s period where light pressure is applied to the DCS probe by hand, aiming to reduce SBF as calculated from the 5-mm detector by ∼50% (based on real-time feedback from instrumentation). As there was some delay in the ${\mathrm{BF}}_{i}$ display, for some cases the 5-mm detector ${\mathrm{BF}}_{i}$ ended up decreasing up to 90%. Pressure is then released afterward and recording continued for 10 more seconds during recovery, totaling to a minute-long measurement.

Hypercapnia measurements were then acquired twice per subject. Two gas supply tanks were used, one with medical air, and the other with a gas mixture of 10% ${\mathrm{CO}}_{2}$, 21% $O_{2}$, and balance $N_{2}$ to ensure that no more than 10% ${\mathrm{CO}}_{2}$ could be delivered to the subject. A flow rate of 10 to 15  l/min was used throughout the entire measurement, and end-tidal $P_{\mathrm{ET}}{\mathrm{CO}}_{2}$ was continuously monitored and recorded simultaneously with the optical and TCD data acquisition (if applicable). Each measurement started with a 1-min medical air baseline period (normocapnia), followed by a 2-min hypercapnic period, and finally another 2-min normocapnic period for recovery, for a total measurement period of 5 min. The fraction of inspired ${\mathrm{CO}}_{2}$ was adjusted as needed throughout the 2-min hypercapnia period to obtain a 10±2  mmHg increase in $P_{\mathrm{ET}}{\mathrm{CO}}_{2}$ from baseline (generally requiring a 5% to 6% inspired ${\mathrm{CO}}_{2}$ fraction).

### 2.2 $P_{\mathrm{ET}}{\mathrm{CO}}_{2}$ Processing Methods

Raw data for $P_{\mathrm{ET}}{\mathrm{CO}}_{2}$ consist of an exported ${\mathrm{CO}}_{2}$ partial pressure timecourse from LabChart at a sampling rate of 1 kHz. To obtain the increase in $P_{\mathrm{ET}}{\mathrm{CO}}_{2}$ throughout the 5-min breathing protocol and filter out respiratory oscillations, we obtained the upper envelope of each measurement and subtracted the average value of this envelope during the baseline from the entire timecourse.

### 2.3 TCD Processing Methods

Raw data from the TCD instrument consist of two timecourses of MCAV in cm/s, one each for the LMCA and RMCA at a sampling rate of 1 kHz. Heart rate and/or respiratory oscillations must be filtered out to obtain a clean MCAV timecourse usable for comparison to DCS data. We first obtained the upper and lower envelopes of each timecourse. Then, a lowpass filter of 0.01 Hz was used on all envelopes; measurements were normalized by dividing the entire timecourse by the average baseline MCAV and converting all values to percent change from baseline. Finally, the upper and lower envelopes from both (if available) L/RMCA were averaged together to produce a single TCD timecourse for each subject.

For subjects who did not have TCD measurements, we computed the expected change in MCAV by calibrating a group average of our existing TCD measurements with a group average of their corresponding $P_{\mathrm{ET}}{\mathrm{CO}}_{2}$ timecourses. The travel time of the exhaled gas through the sampling tube results in a slight time lag of the $P_{\mathrm{ET}}{\mathrm{CO}}_{2}$ timecourse with respect to the TCD curve. This was compounded by a physiological delay in reacting to the hypercapnia. We correct for this by calculating the $r^{2}$ statistic for linear regression between the TCD timecourse and multiple time-shifted $P_{\mathrm{ET}}{\mathrm{CO}}_{2}$ timecourses whose delay time ranged from 1 to 10 s in 1-s increments. The offset time that resulted in the highest $r^{2}$ statistic between the two timecourses was chosen, and the corresponding linear regression model was then used to calibrate the TCD and shifted $P_{\mathrm{ET}}{\mathrm{CO}}_{2}$ data for the 60- to 250-s time interval of the measurement. All subjects chosen with MC fitting based on systemic physiological drift and signal quality criteria (as described below in Sec. 2.4.2) did not have TCD recordings, so expected MCAV values were calculated from the $P_{\mathrm{ET}}{\mathrm{CO}}_{2}$ timecourses.

### 2.4 DCS Processing Methods

The DCS measurements were processed in two primary ways: first, fitting the data using the analytical solution to the semi-infinite diffusion correlation equation, which employs a single-layer, homogeneous model; and second, fitting against a multi-layered MC model of DCS measurements generated by a simulation of photon propagation through tissue. For both methods, experimentally acquired autocorrelation curves from fibers at equal s–d distances were averaged together to make a single autocorrelation at the corresponding s–d separation. Hypercapnia measurements were processed with a 10-s integration time for each data point (autocorrelation curve), and pressure modulation measurements were processed with a 3-s integration time for each data point.

#### 2.4.1 Analytical fitting solution

Autocorrelation curves for each timepoint and each detector distance were fitted for ${\mathrm{BF}}_{i}$ and the coherence factor β using the analytical solution to the semi-infinite diffusion correlation equation:^30^

$$G_{1}(\rho ,\tau )=\frac{3\mu_{s}^{\prime }}{4\pi }[\frac{\exp(-Kr_{1})}{r_{1}}-\frac{\exp(-Kr_{2})}{r_{2}}]$$ (1)

with

$$K^{2}=3\mu_{a}\mu_{s}^{\prime }+\mu_{s}^{\prime 2}k_{0}^{2}\alpha \langle \Delta r^{2}(\tau )\rangle ,$$ (2)

where $G_{1}(\rho ,\tau )$ is the electric field autocorrelation function at a s–d separation ρ and delay time τ, $\mu_{s}^{\prime }$ is the reduced scattering coefficient, $\mu_{a}$ is the absorption coefficient, $k_{0}$ is the wavenumber of light in the medium, α is the probability of scattering from a moving scatterer, ρ is the s–d separation, $r_{1}=(\rho^{2}+z_{0}^{2}{)}^{1/2}$, and $r_{2}={\left(\rho^{2}+{\left(z_{0}+2z_{b}\right)}^{2}\right)}^{1/2}$, with $z_{0}=\mu_{s}^{\prime -1}$, and $z_{b}=1.76/\mu_{s}^{\prime }$ for a tissue index of refraction of 1.37. The expression $\left\langle \Delta r^{2}(\tau )\right\rangle$ is the mean square displacement of the scattering particles—in this case equivalent to red blood cells (RBCs)— at time delay τ, given by

$$\langle \Delta r^{2}(\tau )\rangle =6{\mathrm{BF}}_{i}\tau ,$$ (3)

where ${\mathrm{BF}}_{i}$ is the blood flow index (modeled as a diffusion coefficient as used in the field).^30^ Finally, the normalized intensity temporal autocorrelation function $g_{2}$ is given by

$$g_{2}(\tau ,\rho )=1+\beta {\left(g_{1}(\tau ,\rho )\right)}^{2};\;g_{1}(\tau )=\frac{G_{1}(\tau ,\rho )}{I(\rho )},$$ (4)

where $g_{1}$ is the normalized electric field temporal autocorrelation, β is the coherence factor dependent on the system characteristics, and I is the photon fluence.

In this study, we assume $\mu_{a}=0.015\;{\mathrm{mm}}^{-1}$ and $\mu_{s}^{\prime }=0.85\;{\mathrm{mm}}^{-1}$ for the 850-nm forehead measurements, based on a group average of optical property measurements on several adult foreheads obtained by our group using a frequency-domain NIRS instrument (unpublished data). We then calculated the corresponding hemoglobin concentration (HbT), assuming a 20% volume fraction of fat, 75% volume fraction of water, and a 62.5% oxygen saturation (${\mathrm{SO}}_{2}$), taking the values for each component absorption coefficient from data compiled by Prahl et al.^39^ The calculated HbT and ${\mathrm{SO}}_{2}$ were then used to derive the forehead absorption values for the other wavelengths used. Scattering values for the other wavelengths were derived from the 850-nm $\mu_{s}^{\prime }$ using a power law dependence on wavelength, with a scattering power coefficient of −1.5. For the calf measurements, only the 785- and 850-nm instruments were used to collect the data, and the optical properties were taken from Warren et al.^40^ Table 2 shows the optical properties used for each wavelength.

Each curve was fit over a delay time range of approximately $10^{-6}$ to $10^{-2}\;s$ (covering the entire decay region). The use of only the upper part of the autocorrelation curves as done in previous studies^33^^,^^41^^–^^43^ was considered but appeared to result in excessive fit variation (shown in the Supplementary Material). We obtain timecourses of ${\mathrm{BF}}_{i}$ and β for each detector distance. We have observed β changes during the applied pressure period with pressure modulation, whereas it remains stable during hypercapnia measurements. Therefore, when processing hypercapnia data, to improve ${\mathrm{BF}}_{i}$ fitting stability, we subsequently re-fit the timecourse for ${\mathrm{BF}}_{i}$ values for each detector distance while holding the β value for each distance constant to the median β of the timecourse obtained from the first fit.

#### 2.4.2 Measurement selection

We focused our analysis on several cases that met a number of criteria. To choose measurements with sufficient SNR and data quality, we selected those with a minimum adequate photon count, at least 5k per second per fiber at 30 mm. From there we looked at measurements with two distinct features in the observed responses to the hypercapnia administration: first, a significant (>20%) and persistent increase in superficial ${\mathrm{BF}}_{i}$ (derived from the 5-mm separation measurements), lasting well beyond the end of ${\mathrm{CO}}_{2}$ administration; and second, a long separation response showing a different temporal profile to what was observed in the 5-mm detector, such as an earlier/higher peak, or a decrease during recovery not observed in the short separation detector (measurements in which the long separation timecourse showed the same response as the short separation, or no response at all, likely implied little brain sensitivity even in the long separation channel). Last, in order to potentially benefit from MC-based processing, the long separation response additionally needed to show clear evidence of scalp physiology contamination—this screens out favorable cases in which the long separation timecourse decreased fully despite the persistent increase in 5 mm response, demonstrating good ability to differentiate between the scalp and brain hemodynamics.

#### 2.4.3 Monte Carlo-based fitting

MC forward simulations of light propagation through tissue were performed using the MCX software package developed by Fang et al.^44^ One billion photons were launched per simulation, and relevant data saved from the software simulation included the path length and momentum transfer for each photon for each tissue layer.

**Fig. 1:**
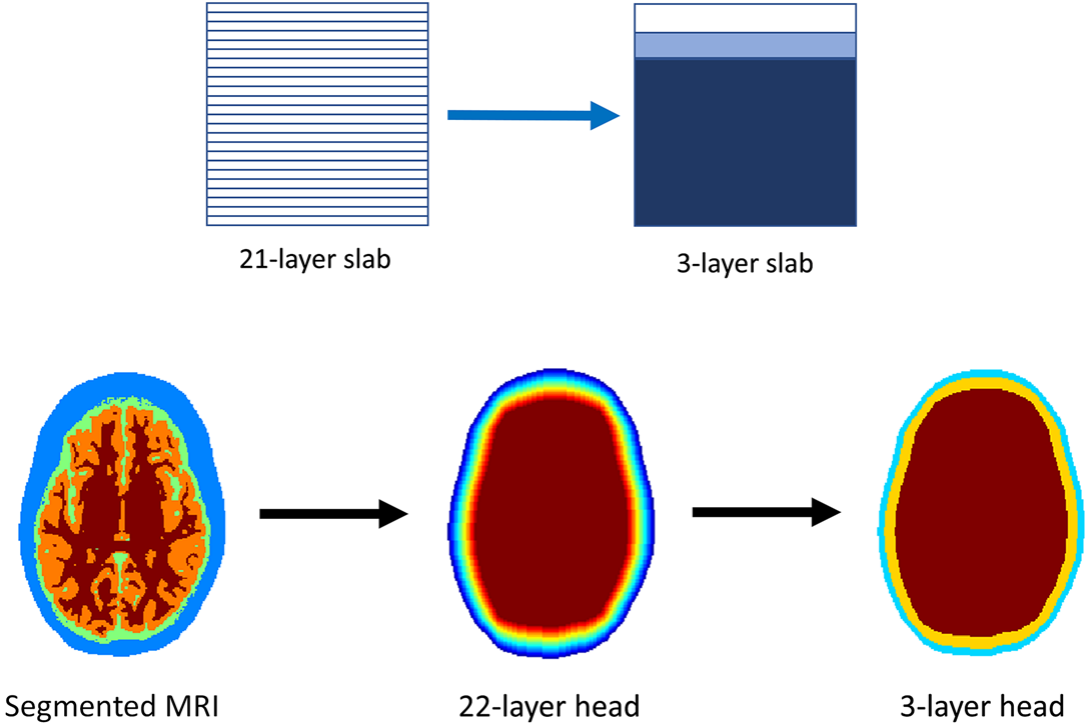
Top row: the 21-layer slab is concatenated into a 2-, 3-, or 4-layer volume in post-processing (3 layer is shown). Bottom row: the segmented MRI is iteratively image-eroded to make a 22-layer head volume and is analogously post-processed into a 2-, 3-, or 4-layer volume.

Two primary volumetric geometries were used for the forward model, both with 1  mm×1  mm×1  mm resolution. The first volume was a 21-layer slab volume, with each layer 1-mm thick. After running the forward simulation, the photon path length and momentum transfer data from 21 layers were then concatenated during post-processing into either two, three, or four total layers, depending on the model tested. Two-layer models represented the extracerebral layers and the brain. Three-layer models replicated the scalp, skull, and the brain; four-layer models denoted the scalp, skull, cerebrospinal fluid (CSF), and the brain. Thicknesses of each layer were adjustable as needed for processing a given measurement. The second volume used for fitting encoded a realistic, head-like model—hereon referred to as the curved model—initially taken directly from a subject’s MRI head scan,^45^ and further iteratively image-eroded to create a final 22-layer head volume, with each layer 1-mm thick (covering the full range of physiologically realistic cortical depths). As with the multi-layer slab volume, the 22 layers were concatenated into two, three, or four total layers as needed. A diagram showing how the slab and curved head models were generated is displayed in Fig. 1.

Measurements were fitted using both the slab and curved models. Two-layer volumes were concatenated such that the extracerebral layer ranged from 1 to 18 mm in 1-mm increments, totaling 18 different MC model versions. Seventy different model versions were used for the three-layer volumes, with scalp thicknesses ranging from 1 to 7 mm and skull thicknesses ranging from 4 to 13 mm in 1-mm increments each; the 70 different models consist of all possible combinations of scalp and skull thicknesses throughout those ranges. Last, thickness combinations for four-layer models were taken from optimal thickness combinations chosen from the three-layer model (selection process is described in depth in Sec. 3.3), with a 1- or 2-mm-thick region removed from the skull layer to create a CSF layer.

For the vast majority of the MC models fittings, scattering was set to $0.85\;{\mathrm{mm}}^{-1}$ for each layer. As an exploration, the impact of using distinct scattering coefficients for each layer was also tested—values used for the scalp, skull, and brain (used with our 850-nm source and taken from Gagnon et al.^18^ as reduced scattering coefficients) were 0.74, 0.81, and $1.16\;{\mathrm{mm}}^{-1}$, respectively. For absorption coefficients, multiple literature reports using our wavelength range of interest for each of the four layers were averaged,^33^^,^^46^^–^^48^ and the values 0.01, 0.033, 0.004, and $0.023\;{\mathrm{mm}}^{-1}$ were used for all MC models. Of note, MC simulation photon history files can simply be reprocessed to take into account changes in absorption, but the MC simulation needs to be re-run to account for changes in scattering as shown below.

Using the photon path lengths and momentum transfer obtained by the MC models, the temporal field autocorrelation function for each tissue layer was calculated as:^30^^,^^32^

$$G_{1}(\tau )=\frac{1}{N_{p}}\overset{N_{p}}{\underset{n=1}{\sum }}\exp(-\frac{1}{3}k_{0}^{2}\overset{N_{t}}{\underset{i=1}{\sum }}Y_{n,i}{\left\langle \Delta r^{2}(\tau )\right\rangle }_{i})\exp(-\overset{N_{t}}{\underset{i=1}{\sum }}\mu_{a,i}L_{n,i}),$$ (5)

where $k_{0}$ is the wavenumber of the light in the medium, $N_{p}$ is the number of photons detected, $N_{t}$ is the number of tissue layers, $Y_{n,i}$ is the total momentum transfer of photon n in layer i, $L_{n,i}$ is the total path length of photon n in layer i, $\mu_{a,i}$ is the absorption coefficient in layer i, and ${\left\langle \Delta r^{2}(\tau )\right\rangle }_{i}$ is the mean square displacement of the scattering particles in layer i, as defined in Sec. 2.4.1. Likewise, the normalized temporal autocorrelation function, $g_{2}(\tau )$, remains the same as in Eq. (4). However, SBF (${\mathrm{BF}}_{i}$ in the topmost layer) was held to the value calculated from the 5-mm detector analytical fit for a given timepoint, and skull blood flow was held to $2\times 10^{-8}\;{\mathrm{mm}}^{2}/s$ (assumed to be 1% of typical head measurement values). As explained in Sec. 2.4.1, β was fitted for at each timepoint, but a second pass fit was done for hypercapnia measurements in which β was held constant for hypercapnia timecourses to the median values for the first pass at each detector.

### 2.5 Estimating Probe-to-Brain Distance

As part of the larger study, some DCS measurements during hypercapnia were acquired in an MRI scanner, where both structural and concurrent functional scans were taken on the subject as validation. While we are not reporting the functional MRI data in this paper, we use the structural MRI scan to estimate extracerebral thickness and compare it to the value predicted by the optimized model in our MC processing.

Multi-echo MPRAGE T1-weighted scans were obtained on the subjects with vitamin E tabs placed on top of the DCS probe as fiducial markers to indicate the optical probe locations. From here, two methods were used to measure extracerebral thickness. The first consisted of loading the MRI DICOMs into the MicroDICOM software package and using the built-in distance tool as a ruler. In the second method, the structural DICOMs were processed into a volumetric segmentation using the MRI Freesurfer image analysis suite.^49^ These volumes were then segmented into four layers—the scalp, skull, CSF, and brain—using methodology presented by Perdue and Diamond.^50^ Finally, the four-layer segmented volume is converted into a mesh-based volume,^51^ with the fiducial marker location preserved throughout the entire process.

The node closest to the fiducial marker location was chosen on the surface of the head, and a normal vector was calculated by averaging the surface normals of all faces containing the node. From there, a line was drawn inward toward the brain along the averaged normal and was incrementally increased in length until the gray matter tissue is reached. This was done for a patch of nodes on the surface surrounding the initial chosen node, up to approximately a 1.5-mm radius, to create a final distribution of calculated probe-to-brain distances.

## 3 Results

### 3.1 $P_{\mathrm{ET}}{\mathrm{CO}}_{2}$ and TCD

**Fig. 2:**
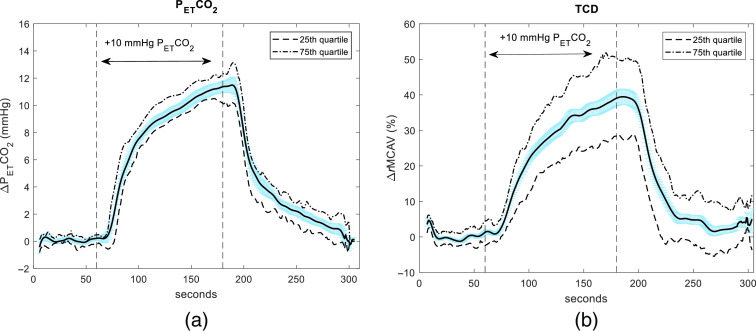
Group averaged $P_{\mathrm{ET}}{\mathrm{CO}}_{2}$ timecourse for all subjects with concurrent TCD measurements, with standard errors in light blue and the quartiles plotted in dotted lines (a), and the corresponding averaged TCD timecourse for the same subjects (b).

Figure 2(a) shows the $P_{\mathrm{ET}}{\mathrm{CO}}_{2}$ group average of the 9 subjects totaling 18 measurements for whom TCD measurements taken concurrently alongside optical acquisitions. Ten measurements had both left and right MCAV timecourses; four had only the left and another four had only the right. Taking the upper and lower envelopes of the total available timecourses resulted in 56 timecourses used for the group average. To remove spikes in the individual timecourses that were otherwise valid, we excluded the highest and lowest data points on a timepoint-by-timepoint basis. An increase of ∼11.5±0.6  mmHg (mean±standard error), a value slightly above our protocol target of 10±2  mmHg, is reached before the sharp decrease starting at 189 s. Accordingly, Fig. 2(b) shows the group average (outliers trimmed) for the TCD measurements corresponding to the $P_{\mathrm{ET}}{\mathrm{CO}}_{2}$ timecourses in Fig. 2(a). The ΔMCAV reaches approximately 39% ±2% above baseline before a sharp drop starting at about 194 s.

**Fig. 3:**
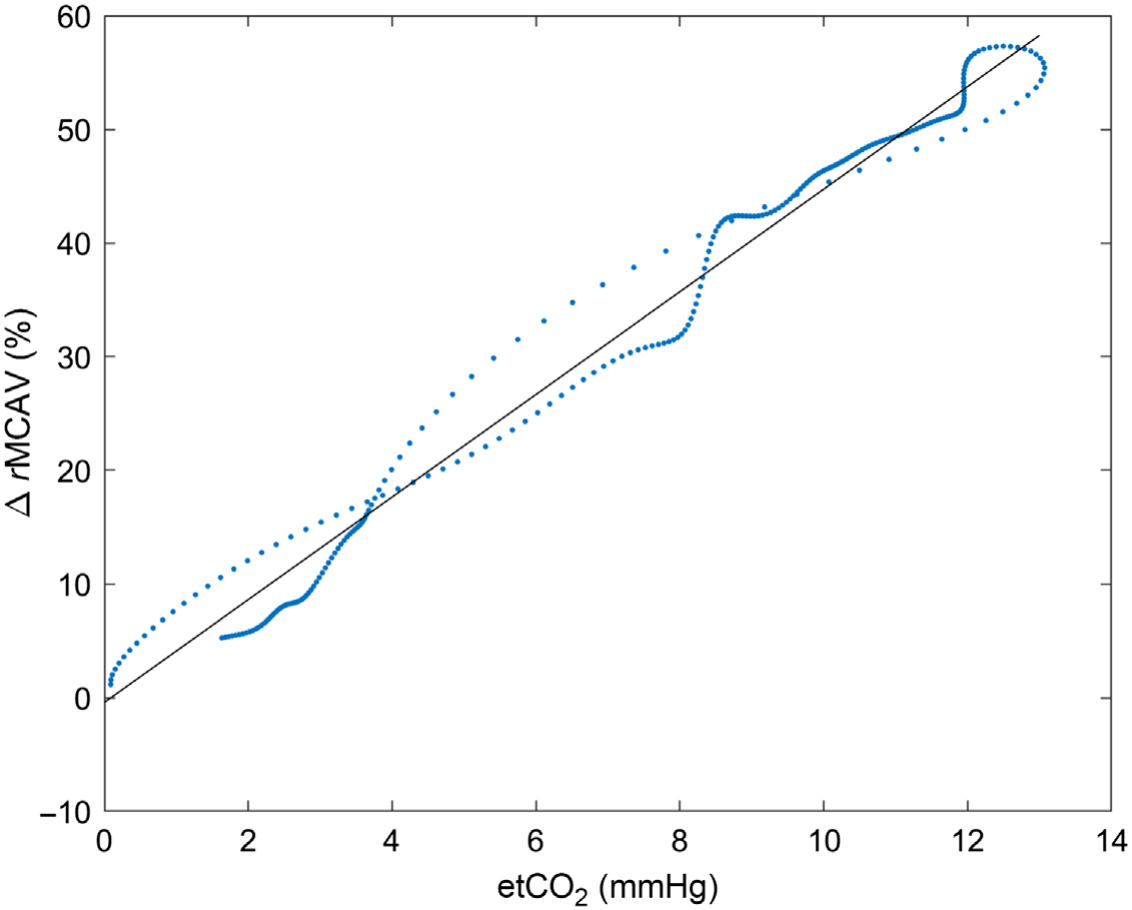
Example ΔTCD timecourse plotted against its corresponding $\Delta P_{\mathrm{ET}}{\mathrm{CO}}_{2}$ timecourse. The linear regression fit is overlaid in blue.

We regressed the individual TCD responses from their respective changes in $P_{\mathrm{ET}}{\mathrm{CO}}_{2}$ for each of the nine subjects. The average slope across the nine subjects was 3.2±0.9 (given as mean and standard deviation); the average intercept was −0.4±4.0; and the average $r^{2}$ value was 0.92±0.07. Figure 3 shows an example plot of the linear relationship between $P_{\mathrm{ET}}{\mathrm{CO}}_{2}$ and MCAV in one subject and shows the relationship between the curves in Fig. 2 for the interval from 60 to 250 s as described in Sec. 2.3. The average $P_{\mathrm{ET}}{\mathrm{CO}}_{2}$ timecourse in this particular case was offset with a time lag of 5 s. Estimation of the TCD timecourses from the ${\mathrm{etCO}}_{2}$ data for the remainder of this study was fitted with the following model: $\Delta \mathrm{eTCD}=3.2\cdot \Delta P_{\mathrm{ET}}{\mathrm{CO}}_{2}-0.4$, where eTCD is the estimated TCD MCAV derived from the $P_{\mathrm{ET}}{\mathrm{CO}}_{2}$ change.

### 3.2 Observed Group-Averaged ${\mathrm{BF}}_{i}$ Changes due to Hypercapnia Using Semi-Infinite Analytical Model

**Fig. 4:**
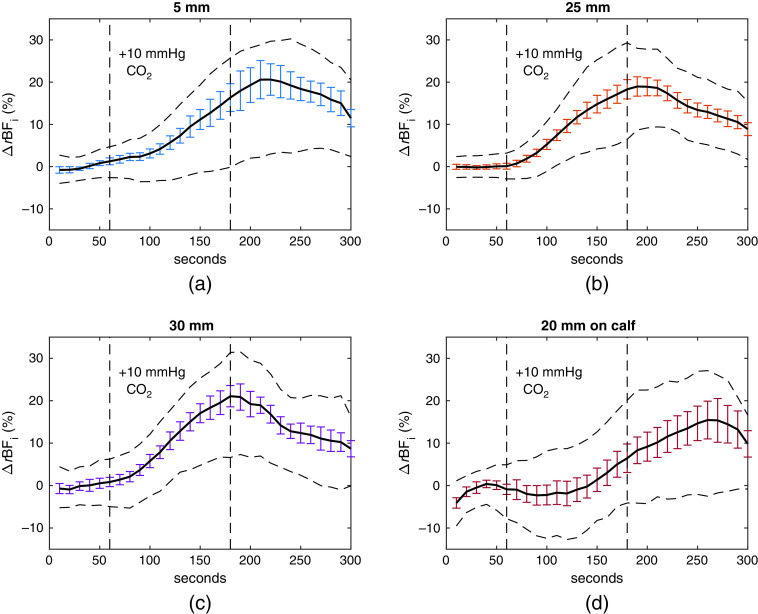
Group average of (a) 5-mm detector, (b) 25-mm detector, and (c) 30 mm detector, totaling 46 measurements. (d) A 28-measurement subset of those that contained a 20-mm probe on the subject calf. The error bars represent standard error, and the dotted timecourses above and below the group average represent the 25th and 75th quartiles, respectively.

Figures 4(a)–4(d) show group averages of the 5-, 25-, and 30-mm s–d separation for measurements during the hypercapnia challenge from subjects for whom data were acquired at all three distances and the baseline ${\mathrm{BF}}_{i}$ (consisting of the 1-min prior to ${\mathrm{CO}}_{2}$ administration) had a coefficient of variation (CoV) of less than 25%. Thirteen out of 59 measurements were excluded due to the CoV criteria. Outliers were excluded at the 5% level on a timepoint-by-timepoint basis again to remove spikes in individual timecourses that were otherwise clean. This resulted in two data points, the highest and the lowest, removed per timepoint for the 5-, 25-, and 30-mm distances, and no data points for the 20-mm distance. Figure 4(d) shows a group average of the subset of these measurements that contained a 20-mm separation probe attached to the subject calf (total of 28 measurements across 10 subjects). We define $\Delta {\mathrm{rBF}}_{i}(\%)$ as $({\mathrm{rBF}}_{i}-1)\times 100$, where ${\mathrm{rBF}}_{i}$ is the ${\mathrm{BF}}_{i}$ timecourse divided by the average value at baseline. The timecourses peak at 220, 190, 180, and 270 s, respectively, reaching increases of 20%±3%, 18%±2%, 20%±2%, and 15%±4%, at 5-, 25-, 30-mm forehead, and 20-mm peripheral s–d separations, respectively. Recovery at 300 s reaches 11%±2%, 8%±1%, 8%±1%, and 10%±2% of baseline.

We consider significant systemic drift to be an observed ${\mathrm{BF}}_{i}$ increase in the 5-mm s–d channel that reached at least 20% during or after hypercapnia. Among the sessions containing all three s–d distances shown in Fig. 4 (where each session for a subject consisted of two hypercapnia measurements), 63% of sessions contained at least one measurement with significant systemic drift. We note that across the larger hypercapnia challenge study, which included subjects with only 5- and 30-mm s–d distances used, a 61% ratio was observed for the same phenomenon.

We extracted the peak times for each individual timecourse used in Fig. 4 and plotted the spread of the peak times for each individual detector distance (see the Supplementary Material). The spreads showed a similar result to the group averages, in that the median of the peak times was earliest for 30 mm, followed by 25, 5, and 20 mm.

### 3.3 Subject 1: Exploration of Various Multi-Layer Monte Carlo DCS Models to Reduce Systemic Physiology Cross-Talk in a Sample Case

Based on the criteria outlined in Sec. 2.4.2, seven total measurements, each from a different subject, were found containing both significant systemic (scalp) ${\mathrm{BF}}_{i}$ change and an observed, distinct long separation increase during hypercapnia that was discernable from the 5-mm response. Four of those measurements had long separation responses returning to within 10% of baseline at the end of the measurement, indicating limited impact of systemic physiology cross-talk. Thus, we performed multi-layer, MC-based fitting on a total of other three measurements.

Since no concurrent TCD data were collected for these measurements, hypercapnia timecourses for all three measurements are shown up to the 250-s mark, as the expected TCD timecourse synthesized from $P_{\mathrm{ET}}{\mathrm{CO}}_{2}$ change using the calibration curve discussed in Sec. 3.1 does not include data past 250 s. In sections below, we first explore several different MC modeling approaches on the first subject measurement: two-layer, three-layer with uniform scattering across layers, three-layer with variable scattering, and four-layer using a range of diffusion coefficients for the CSF. Finally, based on the results from the first subject using the various approaches listed above, we report on the use of the most successful MC model approach for the other two subject measurements presented.

#### 3.3.1 Subject 1: reference analytical fit

**Fig. 5:**
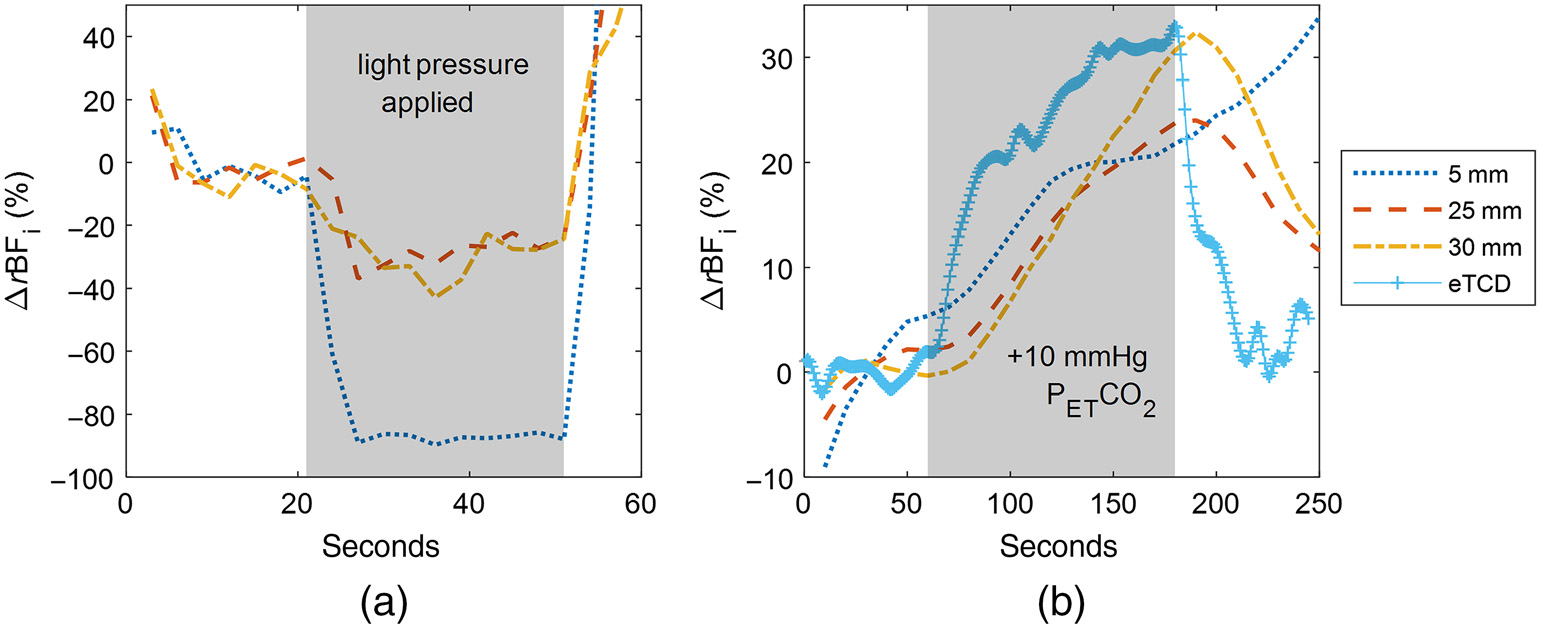
Analytical fitting-based timecourses of ΔrBFi of subject 1 during (a) pressure modulation with light pressure applied approximately between 20 and 50 s from the start, and (b) hypercapnia in terms of the percentage change in the baseline normalized $\Delta {\mathrm{rBF}}_{i}$. Shading indicates the pressure period for (a) and the ${\mathrm{CO}}_{2}$ enriched breathing gas delivery period for (b).

Figure 5 shows the analytical fitting-based timecourse for both the pressure modulation and hypercapnia measurements on subject 1 with expected TCD changes (eTCD) overlaid.

In the pressure modulation timecourse, we observe a 30% to 40% reduction in ${\mathrm{BF}}_{i}$ in the long s–d separations and 85% to 90% decrease in the short separation measurement during the period where light pressure is applied on the probe to reduce SBF. For the hypercapnia measurement, we observe a continuous increase in ${\mathrm{BF}}_{i}$ measured from the 5-mm detector, reaching 33% increase from baseline at 250 s. We note a 23% and 31% increase in the 25- and 30-mm detector timecourses, respectively, both peaking at approximately 190 s; they additionally remain elevated at slightly above 10% at 250 s. The expected TCD timecourse, estimated from the $P_{\mathrm{ET}}{\mathrm{CO}}_{2}$ data, is overlaid; a 32% peak increase at approximately 180 s is reached before a return to baseline around 220 s.

#### 3.3.2 Subject 1: Monte Carlo two-layer (MC-2L) models

**Fig. 6:**
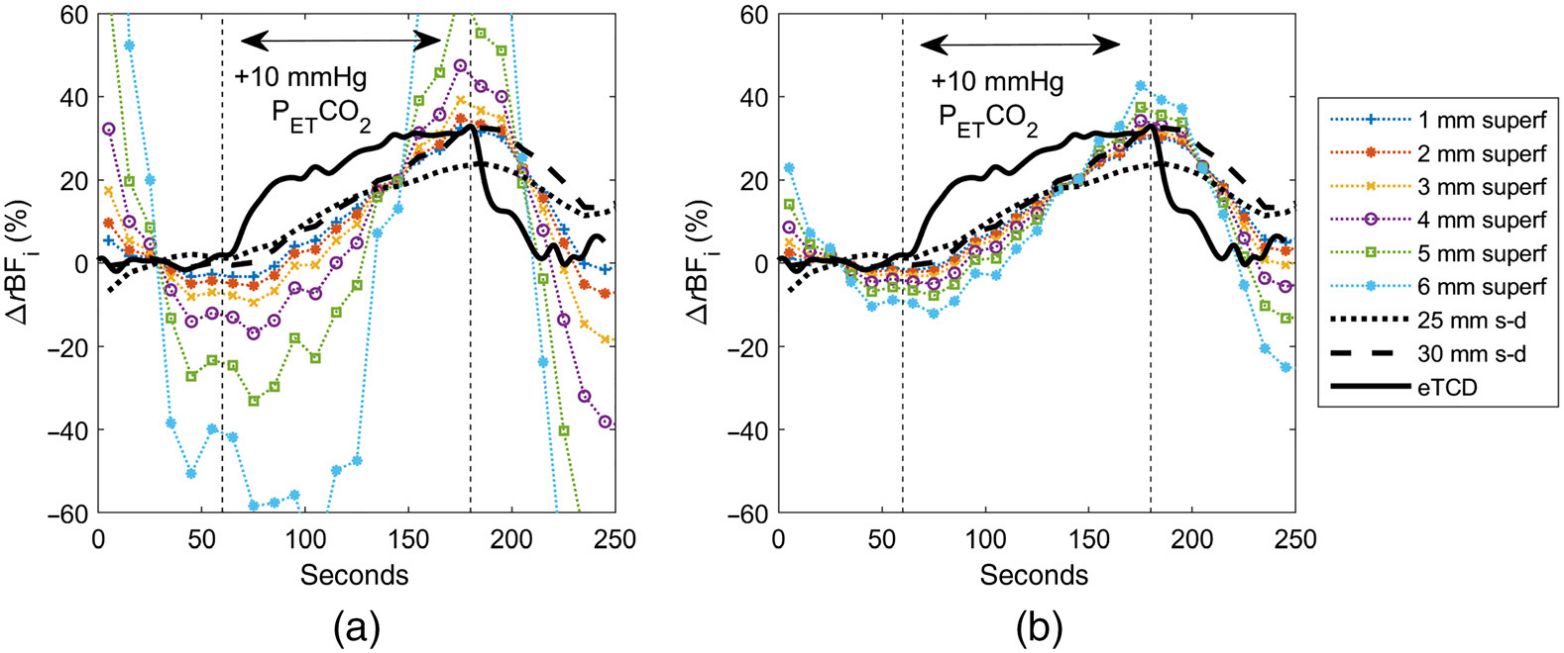
Subject 1 hypercapnia timecourse fitted with (a) MC-2L slab model and (b) curved model overlaid with the analytical fit at 25- and 30-mm separations and eTCD. Only fitting results using MC models with up to 6-mm extracerebral thickness are shown.

As MC methods fit for ${\mathrm{BF}}_{i}$ values for the deep tissue layer as opposed to the analytical method, which fits ${\mathrm{BF}}_{i}$ per detector (described in Sec. 2.4.3), we use the term ${\mathrm{CBF}}_{i}$ for MC-based blood flow approximations. While minimal differences in ${\mathrm{CBF}}_{i}$ timecourse were observed in using just the 5- and 30-mm separation data versus the 5-, 25-, and 30- mm separation data in the MC-based processing algorithm, the pressure modulation timecourse for subject 1 was less noisy with the former option. We therefore show MC-based fits for this subject that were processed using input from just the 5- and 30-mm separations, whereas the MC fitting for the other subjects used inputs from all three s–d distances. Thus, we proceed with MC-based two-layer (MC-2L) slab and curved models to fit the subject 1 hypercapnia timecourse, as shown in Fig. 6. Models with extracerebral thicknesses up to 18 mm were used to fit, but only a subset of them is shown. While a complete return to baseline by 250 s is reached at an unrealistic 1 mm (or 2 mm, for the curved model) extracerebral thickness, the overall response profile does not align well with eTCD. Increasing the extracerebral thickness only pulls the ${\mathrm{CBF}}_{i}$ down to further unrealistic values during the recovery session (shown up to 6 mm, ${\mathrm{CBF}}_{i}$ changes for thicknesses beyond 6 mm become increasingly disproportionate to what is expected). Response differences in the same extracerebral thicknesses between the slab and the curved model are observed. For instance, a 5-mm extracerebral thickness in the slab model decreases to about 80% under baseline by 250 s, whereas the same extracerebral thickness in the curved model only decreases to about 15% under baseline at the same time. In general, for the same extracerebral thickness, the curved model timecourse produces less relative drop in ${\mathrm{CBF}}_{i}$ during recovery than the slab model.

#### 3.3.3 Subject 1: Monte Carlo three-layer (MC-3L) models with uniform scattering across layers

**Fig. 7:**
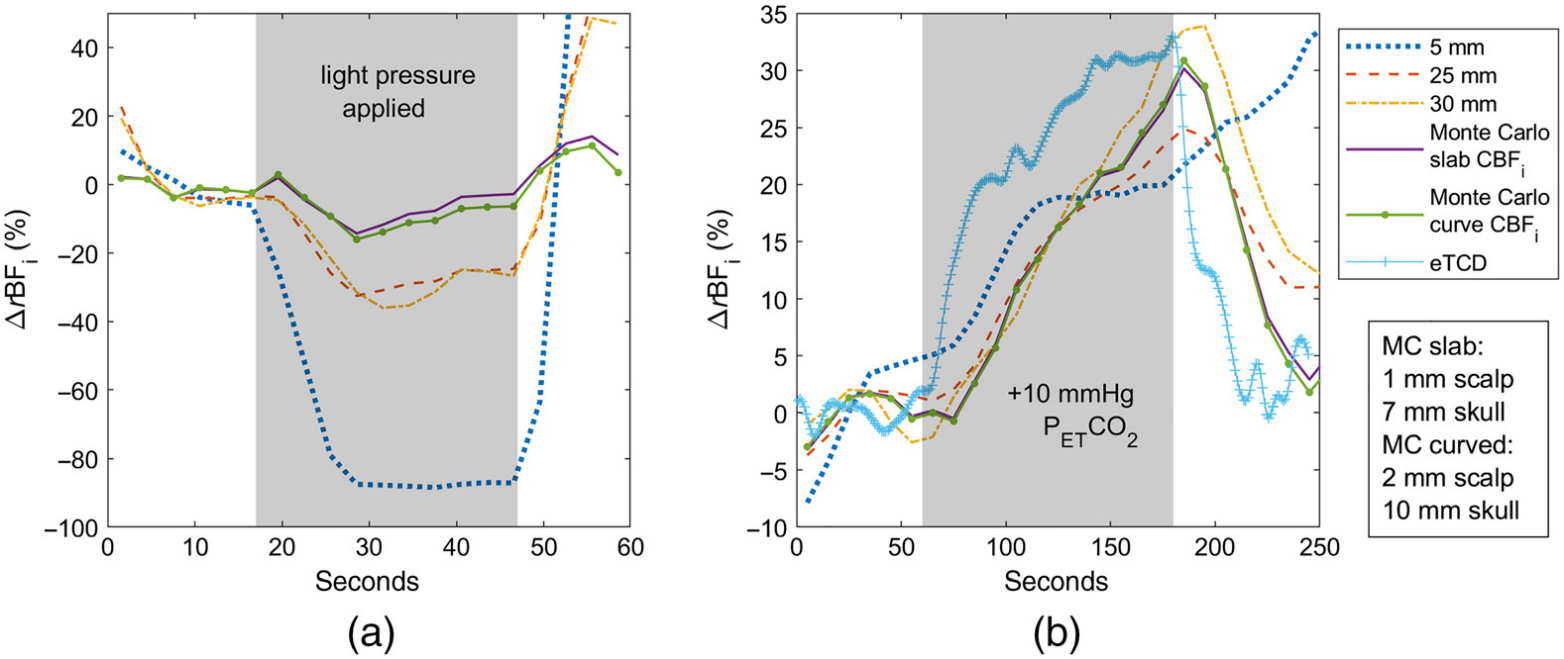
Subject 1 timecourses fitted with MC-3L slab and curved models overlaid with the analytical fit at 5-, 25-, and 30-mm separations and eTCD during (a) pressure modulation and (b) hypercapnia measurements.

Monte Carlo three-layer (MC-3L) fitting results in many more models to select from. To choose the optimal combinations of scalp and skull thickness, we refer to both the $\Delta {\mathrm{rCBF}}_{i}$ timecourses for the pressure modulation measurement and the absolute ${\mathrm{CBF}}_{i}$ timecourses for the hypercapnia measurement. We narrow our selection range to models containing scalp and skull thickness combinations for which ${\mathrm{CBF}}_{i}$ stayed the most constant before, during, and after the pressure modulation period, and also to models for which the brain-to-scalp ${\mathrm{BF}}_{i}$ ratio at baseline was at physiologically relevant levels, between 3 and 8. The final model is chosen where these two criteria converge. Figure 7 shows MC-3L ${\mathrm{CBF}}_{i}$ timecourses for both the pressure modulation and hypercapnia maneuvers in subject 1. For the slab model, a 1-mm scalp and a 7-mm skull thickness were used; for the curved model, a 2-mm scalp and 10-mm skull thickness were chosen. The brain-to-scalp ${\mathrm{BF}}_{i}$ ratio for each model was 3.61 and 4.42, respectively.

In both the MC-3L slab and curved models, ${\mathrm{CBF}}_{i}$ reduction during the pressure modulation reaches a maximum of 16%, significantly less than the reduction observed in the analytical long separation fit. In the hypercapnia protocol, we observe that the peak ${\mathrm{BF}}_{i}$ reached in the MC-3L timecourse occurs 10 s earlier compared to the analytical fit, the MC-3L peak aligning closer to what is shown in the expected TCD timecourse. Last, similar to the eTCD timecourse, the MC-3L timecourse returns to within a few percent of baseline by 250 s, in contrast to the analytical fit, which remains at 10% to 12% above baseline.

#### 3.3.4 Subject 1: Monte Carlo three-layer models with variable scattering in each layer

**Fig. 8:**
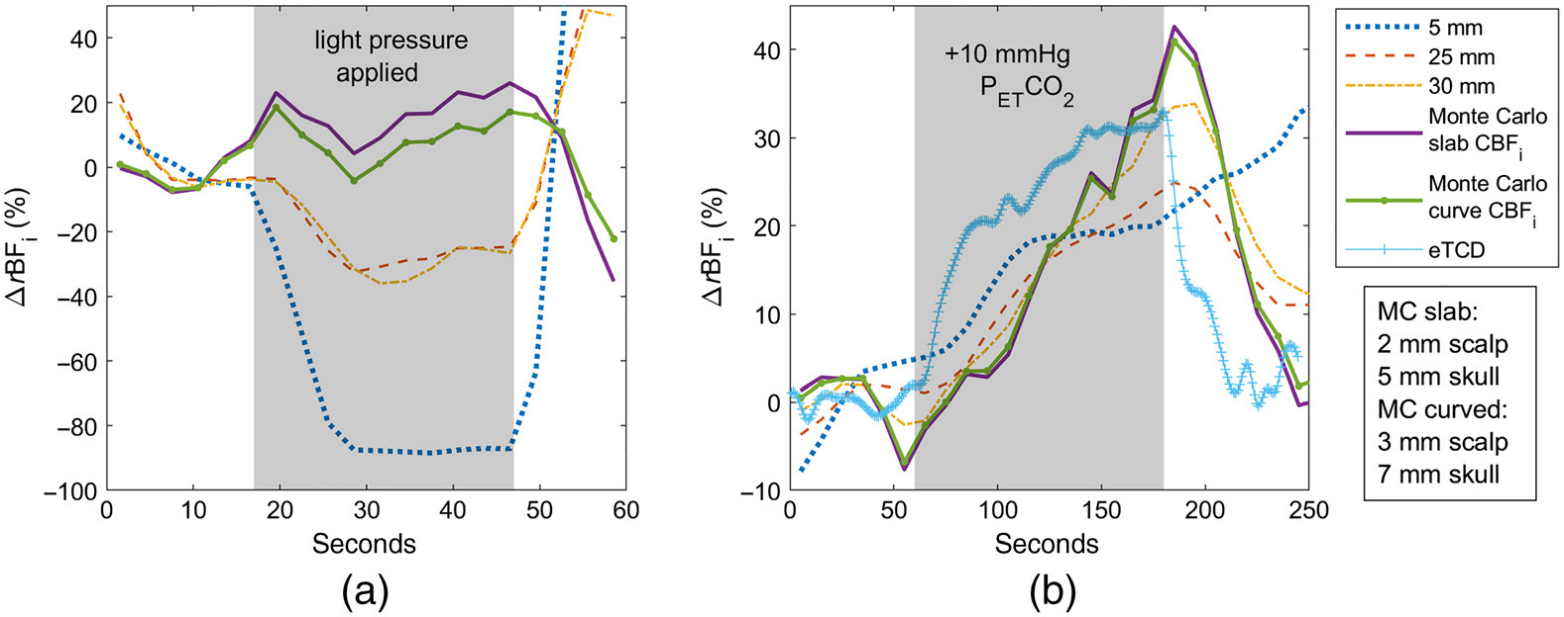
Monte Carlo slab and curved fits for subject 1 for (a) pressure modulation and (b) hypercapnia using variable scattering values across tissue layers.

Figure 8 displays MC-3L fits with scattering values adjusted per layer instead of using a uniform scattering value. As mentioned in Sec. 2.4.3, values for the scalp, skull, and brain used for our 850-nm source were 0.74, 0.81, and $1.16\;{\mathrm{mm}}^{-1}$, respectively. The same criteria for scalp and skull thickness choice were applied with this fit; the slab model used thicknesses of 2 and 5 mm, at a brain-to-scalp flow ratio of 3.75, whereas the curved model used thicknesses of 3 and 7 mm, at a flow ratio of 3.59. We observe a slight overcompensation of ${\mathrm{CBF}}_{i}$ during the pressure period in the slab timecourse, whereas the curved model stays under 20% variation during applied pressure period. However, the responses of the two models are nearly the same during the hypercapnia measurement, with approximately a 10% overshoot in ${\mathrm{CBF}}_{i}$ right after the ${\mathrm{CO}}_{2}$ period ends, but a full return to baseline by 250 s. Given the end results are very similar, this also being the case for the other subjects presented in the paper (results not shown)—we decided to use uniform scattering for the rest of the work.

#### 3.3.5 Subject 1: Monte Carlo four-layer models with variable diffusion coefficients for CSF

**Fig. 9:**
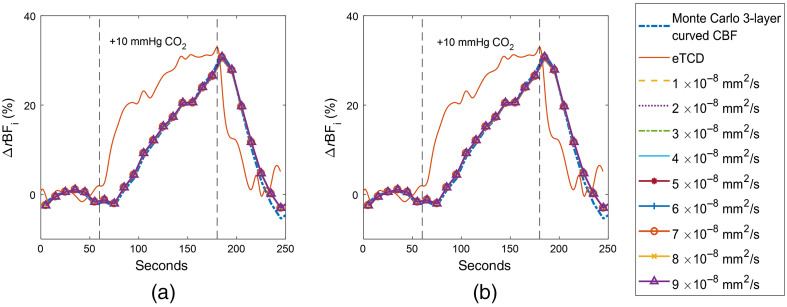
MC curved, four-layer fits with various diffusion coefficients used for the CSF layer for subject 1. A 1-mm CSF thickness was used for (a) and a 2-mm thickness was used for (b).

The final model tested for subject 1 was a four-layer MC curved geometry with uniform scattering across layers, as shown in the timecourse depicted in Fig. 9. The scalp thickness was maintained at 3 mm. One and two millimeters, for Figs. 9(a) and 9(b), respectively, were taken off the 9-mm skull to create a CSF layer between the skull and the brain. We held the diffusion coefficient for the CSF at values ranging from $1\times 10^{-8}$ to $9\times 10^{-8}\;{\mathrm{mm}}^{2}/s$, a span of values approximately centered at the “biological-zero” of ${\mathrm{CBF}}_{i}$ as measured by Busch et al.^52^

Both four-layer curved models using uniform scattering across layers displayed only marginal differences from the three-layer curved fit. We proceed with MC-3L fitting for the remaining subjects.

### 3.4 Subjects 2 and 3: Monte Carlo-Based Three-Layer (MC-3L) Modeling Validation

**Fig. 10:**
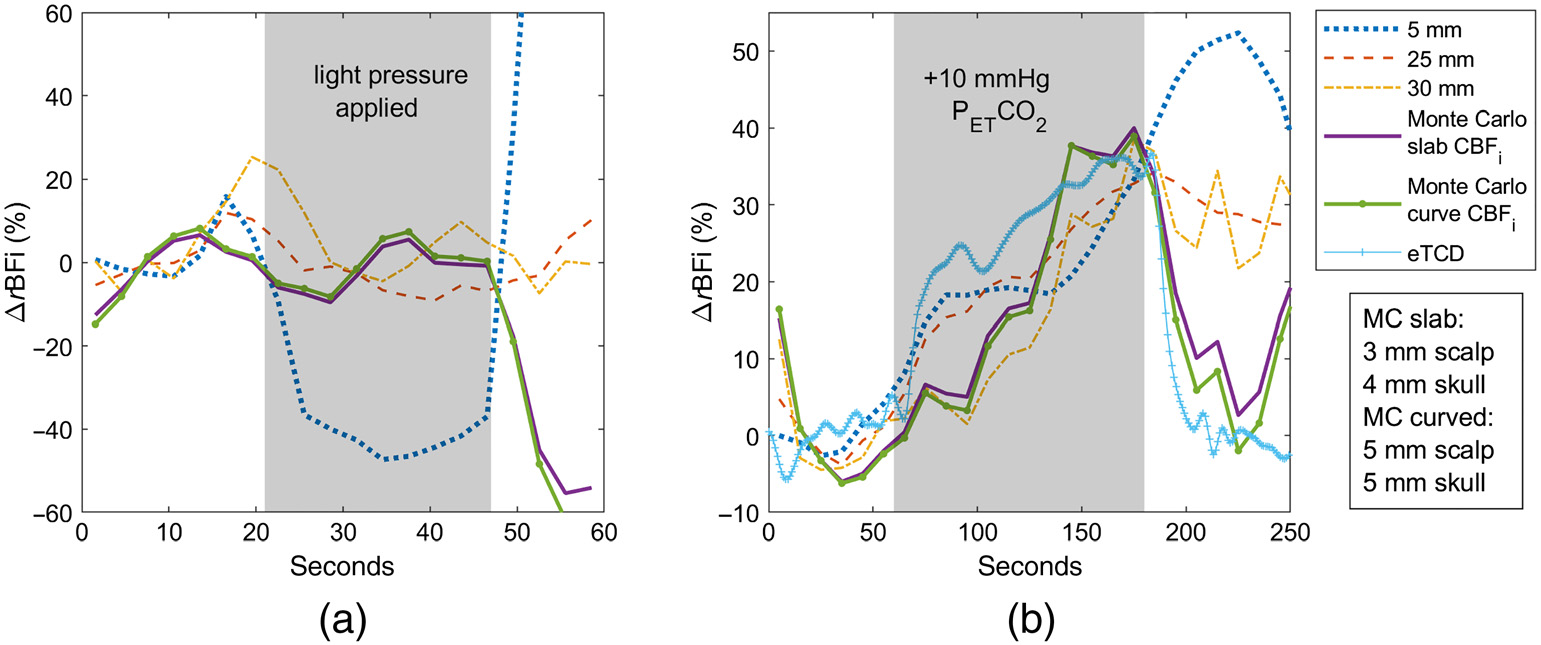
Subject 2 timecourses fitted with MC-3L slab and curved models overlaid with the analytical fit at 5-, 25- and 30-mm separations and eTCD during (a) pressure modulation and (b) hypercapnia measurements.

Figure 10 shows subject 2 MC-3L fits for pressure modulation and hypercapnia timecourses, the latter overlaid with an expected TCD timecourse computed from the $P_{\mathrm{ET}}{\mathrm{CO}}_{2}$ measurement. The slab model had a 3-mm scalp thickness and a 4-mm skull thickness, with a brain-to-scalp ${\mathrm{BF}}_{i}$ ratio of 3.82; the curved model had respective thicknesses of 5 and 5 mm, with the brain/scalp ${\mathrm{BF}}_{i}$ ratio of 3.35.

In the pressure modulation maneuver, we note a 10% decrease in the 25-mm s–d ${\mathrm{BF}}_{i}$ and moderate instability—possibly due to slight motion in the hand pressure—throughout the 30-mm detector timecourse fitted with the analytical model. For the hypercapnia measurement, we see a significant increase of 5-mm s–d ${\mathrm{BF}}_{i}$, peaking a little over 50% of baseline at 230 s. This peak occurs about 47 s later than the eTCD peak. We observe a peak in the 25-mm ${\mathrm{BF}}_{i}$ at 190 s and elevation during the recovery, failing to decrease below 25% by 250 s. We observe a similar continued increase in the post-hypercapnia period for the 30-mm detector at approximately the same percentage.

The MC-3L pressure modulation fits are more stable than what is observed in the 30 mm, although we still see mild variations, up to 10%, comparable to what is seen in the 25-mm analytical fit. In the hypercapnia measurement, both the slab and the curved models peak much closer to the expected TCD curve, albeit a little too early. A return to baseline within a few percentages for the slab and a complete return for the curved model are observed; however, a rise in ${\mathrm{CBF}}_{i}$ after 220 s, similar to what is shown in the 30-mm s–d ${\mathrm{BF}}_{i}$, is also noted for both models. This is likely related to subject motion due to hypercapnia discomfort.

**Fig. 11:**
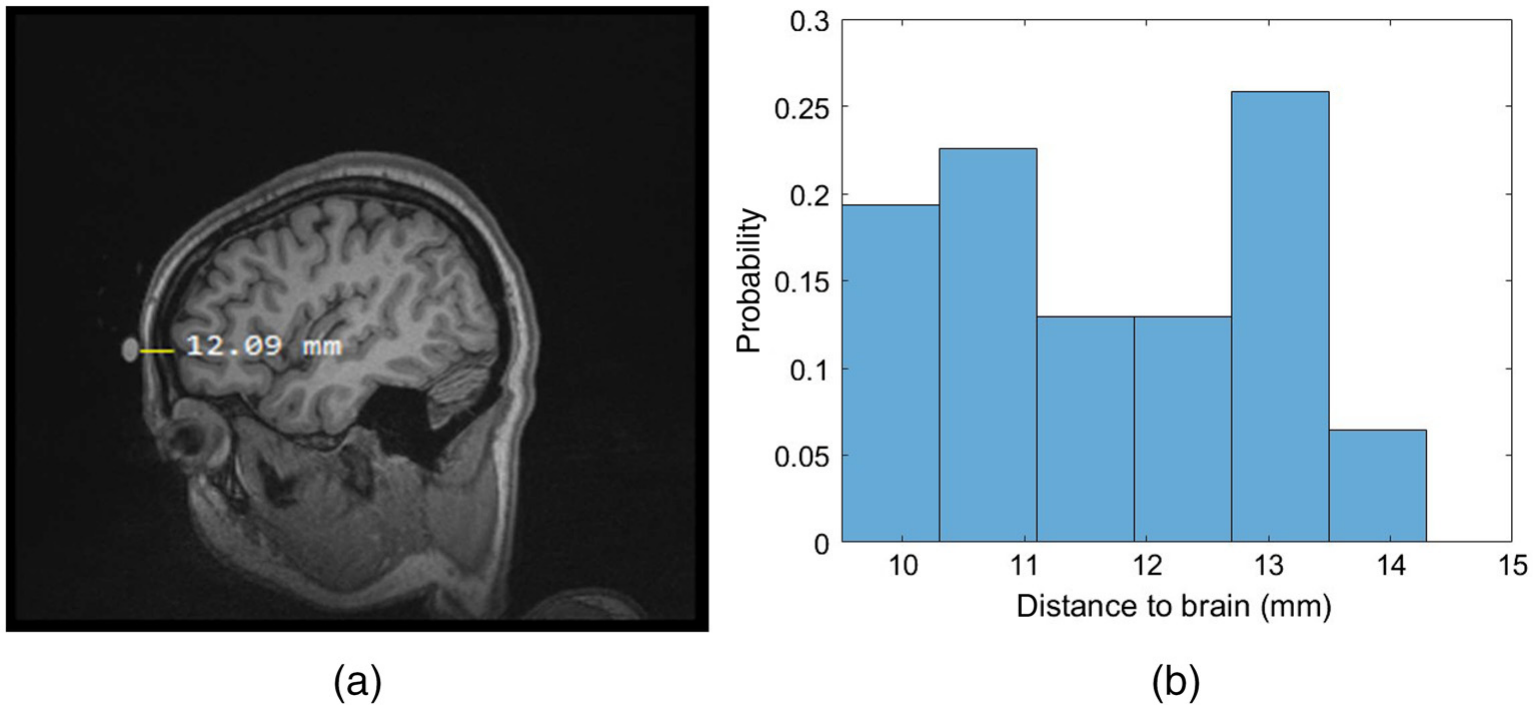
Total extracerebral thickness for subject 2 as measured using a distance ruler on image software (a) and a mesh-based MATLAB function after segmentation (b).

Since MR scans were available for subject 2, we compare the total extracerebral thicknesses used in our MC fitting to what is observed in the MR scan. Figure 11 shows the estimated probe to brain distance as measured using the two methods described above in Sec. 2.5. The optical and MR data for this subject were acquired in one measurement session—that is, the fiducial marker denoting the probe location was preserved between the pressure modulation, hypercapnia, and MR structural scan. We observe a ∼12-mm distance in Fig. 11(a); this falls in approximately the center of the distribution in Fig. 11(b). The thickness combination used for the MC-based slab model is about 5 mm less than what is measured, whereas the MC curved model underestimates the measured extracerebral thickness by 2 mm.

In this subject, we were also able to run MC fitting that used a head segmented volume derived directly from the MRI scan taken as an input to the MC forward simulation. The head volume was segmented into four layers (scalp, skull, CSF, and brain tissue). However, the ${\mathrm{CBF}}_{i}$ results from these fits were very unrealistic (high noise and exaggerated variation), suggesting there may be a systematic bias in the MC modeling of actual anatomy, a potential subject for future investigation.

**Fig. 12:**
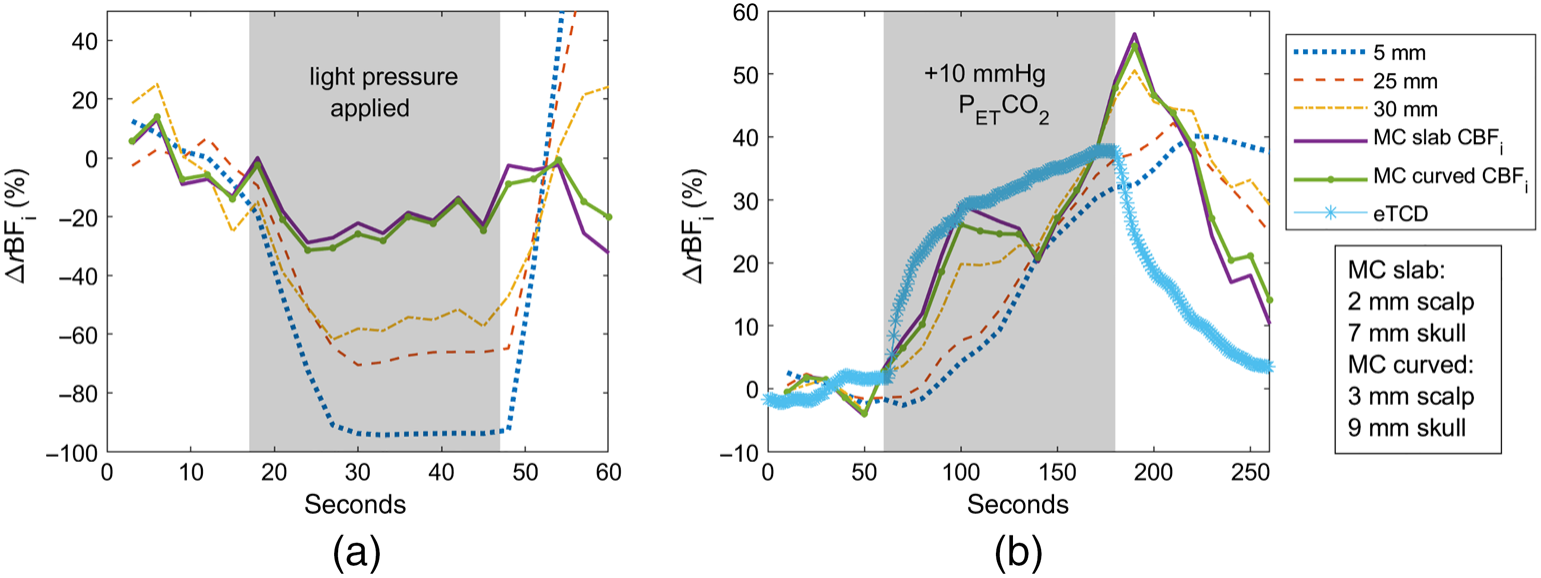
Subject 3 timecourses fitted with MC-3L slab and curved models overlaid with the analytical fit at 5-, 25- and 30-mm separations and eTCD during (a) pressure modulation and (b) hypercapnia measurements.

Last, Fig. 12 displays the MC-3L slab and curved fits for subject 3. A significant drop in the 25- and 30-mm ${\mathrm{BF}}_{i}$ is observed during the pressure period (∼70% and 60%, respectively) in the first plot. We observe ∼30% less of a decrease in ${\mathrm{CBF}}_{i}$ with MC fitting, though it is unable to keep estimated ${\mathrm{CBF}}_{i}$ flat throughout the timecourse. Similarly, while the MC-3L plots do not show ${\mathrm{CBF}}_{i}$ returning to baseline completely at the end of the hypercapnia measurement, they are still able to reduce the ${\mathrm{BF}}_{i}$ elevation by 50% at 250 s compared to the analytical fit. The slab model used a 2-mm scalp and 7-mm skull thickness, whereas the curved model used 3 and 9 mm, respectively. The brain-to-scalp ${\mathrm{BF}}_{i}$ ratio was 3.58 for the slab and 3.12 for the curved model.

## 4 Discussion

### 4.1 Group Response to Hypercapnia

A gradual, significant increase in systemic blood flow due to ${\mathrm{CO}}_{2}$ administration can be observed in both the group average responses shown in 5-mm scalp probe and the 20-mm calf probe. A number of previous studies of hypercapnia-induced CBF changes with DCS have either used only a long separation detector in the DCS probe or an alternate modality for measuring SBF, such as the laser Doppler flowmetry used by Durduran et al.^15^^,^^43^^,^^53^^,^^54^ Our study, as well as recent work by Milej et al.,^55^ shows that measurements without a short separation detector may fail to reveal a possible SBF response that could be driving a significant portion of the observed long separation ${\mathrm{BF}}_{i}$ timecourse. While Durduran et al. reported negligible SBF during the hypercapnia measurement, we note that the data shown were from a single subject, whereas the 5- and 20-mm data shown in our group average span 46 measurements across 18 subjects. As also mentioned in Sec. 3.2, over half of the sessions across our larger hypercapnia study contained at least one measurement with significant (>20% increase) 5-mm response. Selb et al.^33^ have posited the possibility of SBF increase in their DCS measurements during hypercapnia, and several other studies indicate a positive correlation between systemic flow or flow-related parameters and percentage of ${\mathrm{CO}}_{2}$ inhaled.^56^^–^^58^

On the other hand, our cerebrovascular reactivity as measured by TCD is consistent with what Coverdale et al.^59^ showed in a hypercapnia-based validation study between TCD and phase contrast MRI (PC-MRI); they report an approximately 2.5%±2% increase in CBFV per mmHg with TCD and a 3.6%±2%CBFV/mmHg increase with PC-MRI. As shown in Sec. 3.1, we observe a 3.3% increase in CBV/mmHg with TCD. However, we note that Coverdale et al. also reported an 18%±8% higher increase in their calculated arterial CBF versus MCAV during hypercapnia. In our study, the analytical model estimates of ${\mathrm{CBF}}_{i}$ changes from DCS are below TCD changes, likely due to partial volume effects and the fact that a non-negligible minority of our DCS measurements showed little to no ${\mathrm{BF}}_{i}$ increase in the long separation (probably due to low/no brain sensitivity,). The correlates of this apparent lack of sensitivity are still under investigation and a larger sample is likely needed to draw definite conclusions. Moreover, while studies such as Coverdale et al.^59^ and Poulin et al.^60^ are able to derive blood flow from the measured MCAV in TCD, the blood vessels measured by TCD and DCS are not the same; TCD measures the middle cerebral artery, and the estimated velocity is largely determined by the angle between the ultrasound beam and the direction of the blood flow.^61^ DCS, on the other hand, probes microvasculature in the frontal cortex. Apart from the size of the vessels themselves being sources of potentially different CBF values, and Liu et al.^62^ and Duffin et al.^63^ have shown that the cerebrovascular reactivity to increased ${\mathrm{CO}}_{2}$ may not be homogeneous throughout parts of the brain.

Thus, we restrict the comparison of our observed relative ${\mathrm{CBF}}_{i}$ increase during hypercapnia to previous studies that primarily used DCS. Durduran et al.^15^ observed an average of 2.4% increase of ${\mathrm{CBF}}_{i}/\mathrm{mmHg}$ in five adult subjects, similar to our long separation group average shown in Sec. 3.2. Buckley et al.^43^ reported a 49% increase to a 30-min long hypercapnia challenge containing ∼3% inhaled ${\mathrm{CO}}_{2}$, a change significantly higher than what we observe. However, we note that their study was performed on children and not adults, along with a much longer hypercapnia period. Selb et al.^33^ observed an unusually high average ${\mathrm{CBF}}_{i}$ increase of 17% per mmHg; as noted above, this may be a result of a moderate systemic response as shown in their 8-mm separation data. Last, Milej et al.^55^ performed hypercapnia on a group of five subjects and observed approximately a $0.35\;{\mathrm{cm}}^{2}/s$ increase in long separation ${\mathrm{BF}}_{i}$ for a 12-mmHg increase in ${\mathrm{etCO}}_{2}$. While the statistics given in their study cannot be directly compared to ours, the similarity of their observed extracerebral contamination will be noted shortly.

We abstain from directly comparing relative increases observed between our TCD and DCS data. The interpretations we make instead rest on the assumption that because the frontal cortex receives its blood supply from the middle cerebral arteries,^64^ the temporal profile of blood flow in this cerebral region is likely to follow MCAV changes fairly closely. We first note that systemic responses shown in our DCS group averages (increases in 5-mm s–d separation on the forehead and 20-mm s–d separation on the calf, as discussed earlier) display distinct temporal curves and peaks than what is seen in our TCD group average. In particular, the systemic response peaks begin a gradual decrease approximately 30 and 90 s in the 5- and 20-mm s–d separations, respectively, after the end of the ${\mathrm{CO}}_{2}$ administration; the MCAV response as shown in TCD group average peaks drops sharply within 10 s after ${\mathrm{CO}}_{2}$ administration is stopped. Additionally, MCAV returns to baseline within 90 to 120 s at the end of hypercapnia, whereas the 5-mm and 20-mm response remains elevated 10% to 15% above baseline at 300 s. This elevated ${\mathrm{BF}}_{i}$ response during recovery is also observed in 25- and 30-mm data. The 25-mm timecourse peaks earlier than the 5-mm (190 versus 210 s, closer to what we see in the TCD data) and recovers several percentages nearer to baseline at the end of the measurement, but we do not observe the full recovery as shown in TCD. The 30-mm s–d separation, probing deeper and thus containing more signal from the brain, peaks even sooner than the 25-mm curve at approximately 180 s, at the end of hypercapnia. Even so, it still recovers by about the same amount as the 25-mm distance at 300 s. As such, we interpret the group average ${\mathrm{BF}}_{i}$ results as evidence of a systemic response to hypercapnia contaminating results shown in the long-separation detector timecourses. Milej et al.^55^ demonstrate a similar phenomenon, where a lasting elevated response in the SBF is shown to contaminate the long separation ${\mathrm{BF}}_{i}$, and was confirmed via repeating the hypercapnia challenge with a tourniquet tightened over the probe to suppress SBF. They estimated the extracerebral contamination level to be approximately 48%±18% at 3 cm.

As mentioned above, significant scalp perfusion increases in our study were observed in a little over half the measurements. These measurements showed a wide range of scalp reaction magnitudes. The degree of scalp contamination in the long separation ${\mathrm{BF}}_{i}$ will likely vary from subject-to-subject depending on factors, such as extracerebral thickness, amount of scalp reaction, and signal-to-noise ratio in the long separation signal. This phenomenon should be taken into account for future studies using hypercapnic validation and most importantly for studies using DCS to measure brain blood flow changes in adults clinically.

### 4.2 Comparison of Monte Carlo Modeling to Previous Scalp Reduction Approaches

In the NIRS field, short-separation regression approaches have been extensively studied;^21^^–^^26^^,^^65^^,^^66^ however, for DCS, the non-linear nature of how the blood flow affects the different parts of the autocorrelation curve makes it less suitable for short separation regression. Thus, post-processing scalp physiology contamination reduction methods for DCS usually rely on a layered version of the analytical model to fit for ${\mathrm{CBF}}_{i}$.^18^^,^^27^^,^^28^ Our technique can be considered as an MC-based analog of Baker et al.’s analytically derived pressure modulation algorithm,^29^ applied alongside hypercapnia measurements as further model validation. The primary advantages of MC-based modeling are the numerical stability and, as Li et al.^32^ and Shang et al.^67^ have shown, the ability to accommodate realistic geometries as inputs into the forward simulation. As the development of MC photon propagation simulations becomes more sophisticated, MC modeling would allow us to be able to incorporate more nuanced physiological parameters into DCS fitting. This could include investigating the effect of probe location with respect to cortical folds or mapping DCS sensitivity across the entire forehead. Overall, data from MC simulations need not be restricted to *in-silico* validation of analytical models but can be used as inputs to fit *in-vivo* measurements themselves, particularly as MC methods are becoming increasingly faster and more accurate.^44^^,^^68^

### 4.3 Qualifications of Successful Monte Carlo Enhanced ${\mathrm{CBF}}_{i}$ Quantification; Limitations of the Technique

We observe that pressure modulation, when used in conjunction with reasonable physiological estimates of brain-to-scalp ${\mathrm{BF}}_{i}$ ratio and scalp/skull thicknesses, has the potential to help guide optimal models for MC fitting on a case-by-case basis. However, the maneuver may also serve as an indicator of brain sensitivity—measurements in which the long separation ${\mathrm{BF}}_{i}$ shows equal or comparable decrease to the short separation ${\mathrm{BF}}_{i}$ may indicate little or no cerebral sensitivity. Ideally, this should be checked in real time when setting up the measurement, and the DCS probe moved as needed to minimize the relative drop in the long separation measurement compared to the short separation. We interpret subject 3 as an example of this; the long separation ${\mathrm{BF}}_{i}$ decreases significantly more during the pressure period as compared to the other two subjects. Correspondingly, the MC fits for this subject are unable to extract a full recovery to baseline at the end of the measurement. Like subject 3, baseline levels of sensitivity for future subjects may be inferred through protocols, such as pressure modulation or a hypercapnia challenge.

The criteria previously discussed in Sec. 2.4.2 serve as qualifications outlining the extent to which our study of multi-layered MC-based fitting can remove extracerebral influence from ${\mathrm{CBF}}_{i}$. As a brief investigation (results not shown), we performed MC fits on subjects from the protocol with little to no evidence of brain sensitivity in the measurements, i.e., the long separation response did not differ much from the short in either the pressure modulation or hypercapnia measurements. We observed that the MC results were at best the same as the analytical fits; model geometries that showed reasonable brain-to-SBF ratios or increased ${\mathrm{CBF}}_{i}$ during hypercapnia sometimes contained much noisier timecourses (presumably due to model mismatch). Analogously, we conducted MC modeling on measurements that already showed high levels of brain sensitivity in the analytical fit, i.e., significantly higher and timelier increase during hypercapnia and nearly full return to baseline in the long separation. The MC timecourses for these measurements matched the analytical fits. With the current resolution and accuracy in MC photon simulation, fitting DCS data using MC modeling seem to prove most advantageous under specific conditions, where the long separation analytical fit is showing evidence of both brain sensitivity and cross-talk from systemic physiology (verified with short-separation measurements).

Last, we acknowledge the computational limitations imposed by the use of the MC-based models. The two computationally intensive steps are the MC simulation itself, and the post-processing of MC history files to generate autocorrelation curves. With GPU acceleration, the MC simulation for 1 billion photons can completed within a few minutes (and is getting faster every year). Post-processing MC history files to obtain $g_{2}$ curves for given ${\mathrm{BF}}_{i}$ values for each layer take on order of 50 to 100 ms in MATLAB if double-precision GPU acceleration is available or about 5 times longer using CPU only. Once the MC geometry is selected data can be fit in real time on a high end workstation computer as long as scattering is assumed to remain constant (this allows the reuse of the MC history file without re-running the simulation). However, there is a preparatory phase, in which data from one or more pressure modulation maneuvers are processed using a variety of geometries (varying scalp and skull thickness as explained in Sec. 2.4.3)—this could take half an hour or so on today’s hardware using MATLAB code. With highly optimized dedicated code, we believe the preparatory phase can be reduced to a few minutes, after which real-time MC-based monitoring will be available, e.g. if the goal is to offer brain perfusion monitoring in a clinical setting.

### 4.4 Monte Carlo Geometry Choices

As seen in subject 1, two-layered MC-based models appear to fail—in particular, increasing extracerebral thickness closer to what is physiologically accurate leads to overcompensation and a sharp decrease in ${\mathrm{CBF}}_{i}$ during recovery period post-hypercapnia, and no thickness setting follows the TCD measurement temporal profile effectively. The only two-layer models that appear to have reasonable post-hypercapnia recovery responses have 1-2 mm extracerebral thicknesses, which are too thin to be considered realistic. The three-layered models containing a constant diffusion coefficient value for a 4- to 10-mm slice—in other words, with a layer representing the solid slice of the skull—between the probe and the brain succeed in revealing more accurate ${\mathrm{CBF}}_{i}$ responses despite contrasting superficial influence at much more reasonable extracerebral thicknesses. Disregarding the presence of low blood flow in the skull renders the two-layer models as highly unrealistic, contributing to their failure. Using varying scattering coefficient values across the three-layers in the MC-3L model seems to give a similar result as to their respective homogeneous scattering models, though at different scalp and skull thickness combinations. This is expected, as adjusting the scattering and adjusting the superficial layer thickness can both be interpreted as modifying sensitivity to the brain; changes in the former should result in changes to the latter, and vice versa. Given the substantial uncertainty regarding the actual scattering properties of inner tissue layers, the use of uniform scattering values appears warranted in DCS measurement analysis. Last, adding a 1- to 2-mm extra layer for CSF and using a four-layer model seems to have little impact on the final results.

We observe similar responses in the slab and the curved models in both pressure modulation and hypercapnia timecourses after tuning. In all subjects, the difference in the timecourses between the two geometries is close to negligible; however, the optimized slab extracerebral thickness combinations across subjects fall a few millimeters short of their respective curved thickness combinations. Comparing these values to the probe-to-brain distances measured on the structural scan for subject 2 reveals a closer match to the curved extracerebral distances than to the slab. In situations where either the scalp, skull, or total extracerebral thickness can be acquired and subsequently used as an input for model tuning, a curved geometry may be a more accurate and useful model. In other applications where this type of anatomical information is unknown and may never be acquired, the slab and curved models can be seen as functionally equal. With either case, we note the importance of model tuning in MC-based fitting or similar methods where the superficial layer thicknesses are adjusted over a range of values. Some *a priori* or other objective criteria—in this case pressure modulation and the analytical brain-to-scalp ${\mathrm{BF}}_{i}$ ratio—must be used to guide the model to the correct scalp and skull thicknesses. Future research or clinical applications of DCS will involve situations where the expected CBF response cannot be predicted or is unknown; these circumstances necessitate some objective methodology to choose the most accurate model out of all possible scalp and skull thicknesses.

While minor differences are observed in the slab and the curved geometry fits, both can do several things. First, as seen in all subjects, they can provide a more stable ${\mathrm{CBF}}_{i}$ during the pressure modulation maneuver than what is seen in the analytical long-separation fit. Second, the MC models can bring ${\mathrm{CBF}}_{i}$ closer to baseline in post-hypercapnic recovery when superficial contamination is observed in the analytical long-separation fit. Third, as seen especially in subject 1, MC models can substantially remove of scalp influence to the temporal curve of the fit, i.e., they can remove a time lag caused by the scalp flow increase in the DCS ${\mathrm{CBF}}_{i}$ estimate peaking and then decreasing.

## 5 Conclusion

We used DCS and TCD to measure hypercapnic responses in adult human subjects. Comparing results from the two modalities reveals a systemic blood flow increase to hypercapnia, which has the potential to contaminate the long separation ${\mathrm{BF}}_{i}$ when the DCS data are fitted with the semi-infinite homogeneous analytical model. Using multi-layered, MC-based fitting models, combined with a pressure modulation algorithm to guide model choice, shows improvement over the traditional analytical fit in removing extracerebral contaminants. A three-layer model with a constant diffusion coefficient for the skull is likely needed; a curved, head-based model may result in more accurate physiological parameters than a flat slab-based model, but both geometries show comparable improvements in removing superficial influence versus the analytical fit. Future work will continue exploration of more complex and variable multi-layered MC-based models, including neuro-imaging-driven subject-specific models, along with their potentials and limitations.

## Code, Data, and Materials Availability

Data supporting the results reported in the paper can be requested by contacting the corresponding author.
