# Erratum: Improved accuracy of cerebral blood flow quantification in the presence of systemic physiology cross-talk using multi-layer Monte Carlo modeling

**DOI:** 10.1117/1.NPh.9.1.019801

**Published:** 2022-02-17

**Authors:** Melissa M. Wu, Suk-Tak Chan, Dibbyan Mazumder, Davide Tamborini, Kimberly A. Stephens, Bin Deng, Parya Farzam, Joyce Y. Chu, Maria A. Franceschini, Jason Z. Qu, Stefan A. Carp

**Affiliations:** a Massachusetts General Hospital, Harvard Medical School, Optics at Athinoula A. Martinos, Center for Biomedical Imaging, Department of Radiology, Charlestown, Massachusetts, United States; b Massachusetts General Hospital, Harvard Medical School, Department of Anesthesia, Critical Care and Pain Medicine, Boston, Massachusetts, United States

## Abstract

The erratum corrects an error in Equation 1 of the published article.

This article [*Neurophotonics* **8**(1), 015001 (2021) doi: https://doi.org/10.1117/1.NPh.8.1.015001] was originally published on 11 July 2021 with an error in Equation 1. The equation should have had negatives in both of the exponent expressions [i.e., exp(−*Kr*_1_) and exp(−*Kr*_2_)].

The negatives were added to the exponent expressions to correct the equation and the article was republished on 4 February 2022.

