# Supplementary material for: Improved accuracy of cerebral blood flow quantification in the presence of systemic physiology cross-talk using multi-layer Monte Carlo modeling
Source: Neurophotonics. 2021 Jan 1;8(1):015001. doi: 10.1117/1.NPh.8.1.015001 (PMC7779997; doi:10.1117/1.NPh.8.1.015001)
Supplement: Supplementary file 1 [file NPh_008_015001_SD001.pdf]

Improved accuracy of cerebral blood flow quantification in the presence of systemic physiology cross-talk using multi-layer Monte Carlo modeling

Melissa M. Wu, Suk-Tak Chan, Dibbyan Mazumder, Davide Tamborini, Kimberly A. Stephens, Bin Deng, Parya Farzam, Joyce Yawei Chu, Maria Angela Franceschini, Jason Zhensheng Qu, Stefan A. Carp

Supplementary Material

We compared the effect of adjusting the g_2 fitting range on our DCS data. Selb et al.[33] have shown that restricting the fitting range to earlier delays results in increased cerebral contribution to the BFi signal. Figure S1 shows the DCS group averages from section 3.2 overlaid with the same data fitted only using the early delays. For the latter, fitting was confined to where the fitted g_2 was no less $1 + \beta/2$, where β was the median value of the first fitting pass as described in section 2.4.1. We observe that the early delay fits result in increased cerebral sensitivity seen at the peak of the hypercapnia period in the 25 and 30 mm s-d distances. However, this comes at the expense of higher noise, particularly at the 30 mm distance.

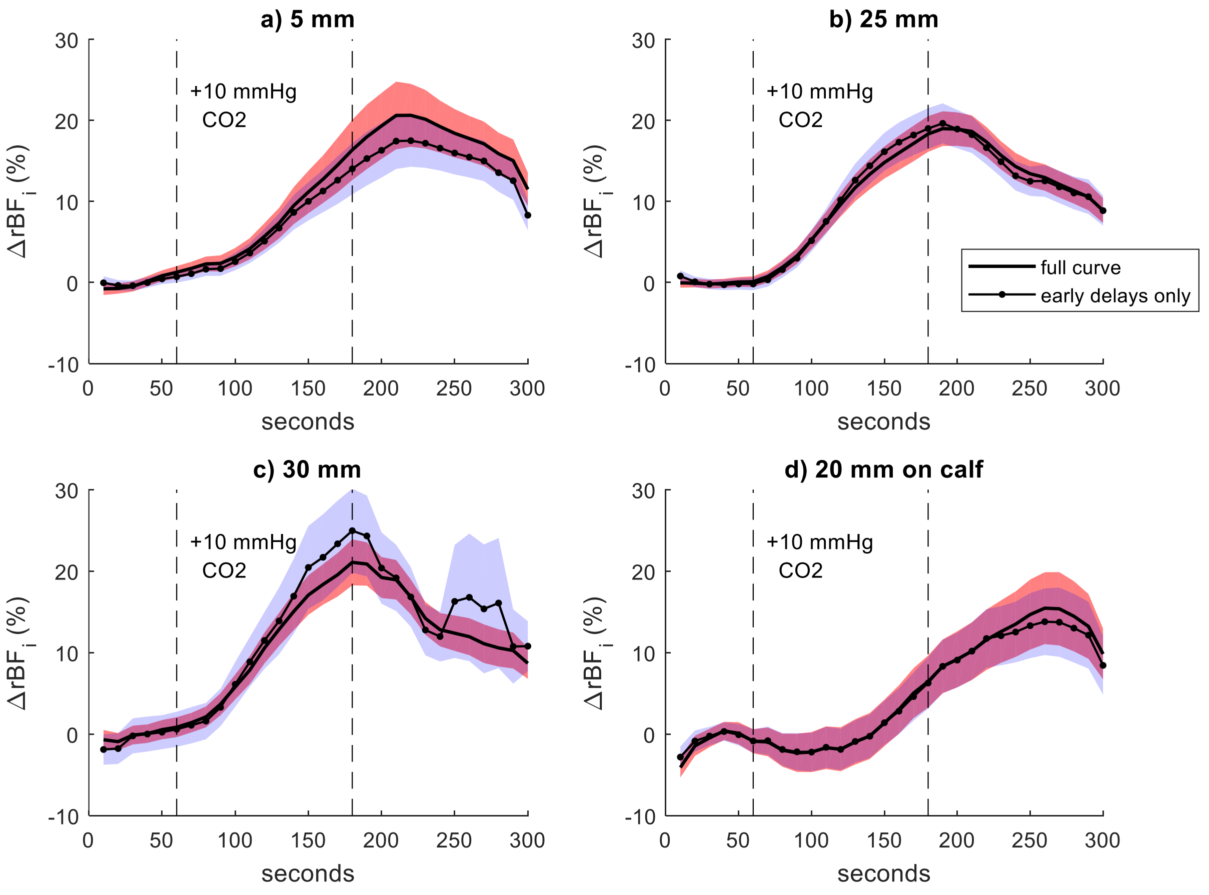

Figure S1. BF_i timecourses where the full g2 curve was fit (solid black line) versus where just the early delays were fit (dotted black line). The standard error range for the full and the early fits are shown as the shaded dark orange and the shaded light blue area, respectively.

We investigated the variability of the BF_i peak times during the hypercapnia measurements in our DCS group average (section 3.2, figure 4). For each individual timecourse, we took the time at which the maximum BF_i value was reached for all source-detector distances. Figure S2 shows the spread of these peak times across all measurements. We observed that similar to the DCS group average, the peak times for the 5 mm forehead and 20-mm peripheral s-d distance channels tend to occur both substantially after the hypercapnia has ended, as well as after the peak times for the 25- and 30-mm distances.

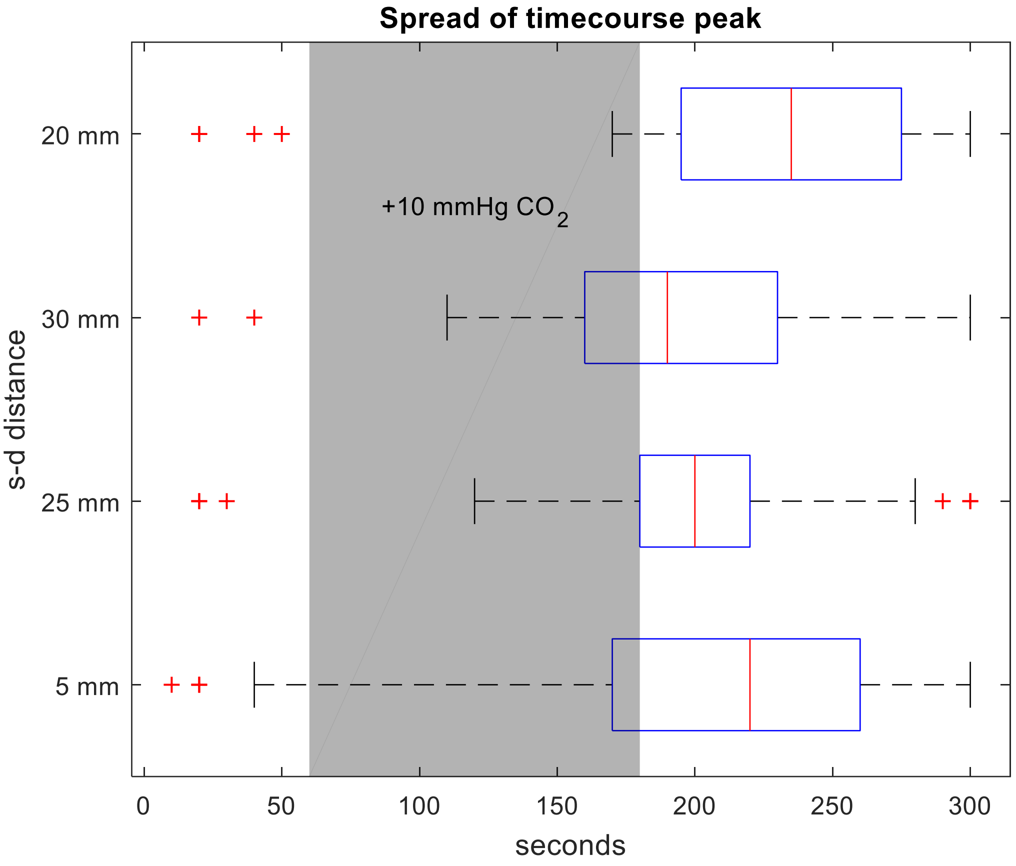

Figure S2. A box-and-whisker plot showing the time at which the maximum BFi value was reached for each detector during the hypercapnia challenge. The data includes all measurements used in the DCS group average.
